# Postharvest warm conditioning associates with HMGR abundance, squalene accumulation, and MAPK/phosphatase transcriptional responses in *Camellia oleifera*

**DOI:** 10.3389/fpls.2026.1804924

**Published:** 2026-04-23

**Authors:** Jianwen Wu, Rong Qin, Yingying Chen, Jihua Guan

**Affiliations:** 1 Guangxi Forestry Research Institute, Guangxi Laboratory of Forestry, Nanning, China; 2 Guangxi Gaofeng State Owned Forest Farm, Nanning, China

**Keywords:** *Camellia oleifera*, HMGR, MAPK signaling, postharvest warm conditioning, protein phosphatase, squalene accumulation

## Abstract

**Introduction:**

Fully mature Camellia oleifera fruits undergo a brief postharvest warm-conditioning step that can markedly affect oil quality and squalene levels, yet its early molecular basis remains unclear.

**Methods:**

We integrated transcriptomics, UPLC–MS/MS metabolomics, targeted biochemical assays, and RT–qPCR to profile seed-kernel tissues collected from fruits incubated at 35 °C and 95% RH for 0, 12, and 24 h.

**Results:**

Warm conditioning increased squalene content and HMGR abundance in seed-kernel tissues. RNA-seq revealed broad transcriptional reprogramming, including coordinated changes in mevalonate pathway-associated genes and multiple phosphorylation-related components. Among four HMGR transcripts, HMGR-2 showed the highest baseline abundance, whereas HMGR-1 showed the strongest relative induction at 12 h, as confirmed by RT–qPCR. In parallel, MPK3/MPK6-like kinases and PP1/PP2A subunits were transcriptionally responsive to the treatment.

**Discussion:**

These multi-omic and biochemical data define an early postharvest warm-conditioning response associated with increased HMGR abundance and squalene accumulation, while providing candidate genes and pathway context for future functional validation.

## 1 Introduction

*Camellia oleifera* Abel. (*C. oleifera*) is a major woody edible oil crop cultivated primarily in China (Zhu et al., 2020; Li et al., 2022). Its seeds are oil-rich, and the resulting camellia oil is valued for its high proportion of unsaturated fatty acids and multiple minor bioactive constituents (Zhu et al., 2020; Li et al., 2022; Zhong et al., 2026). Beyond its role as a premium edible oil, *C. oleifera* also supports cosmetic, medicinal, and by-product utilization chains, making postharvest fruit handling directly relevant to both product quality and industrial value. In commercial practice, freshly harvested fruits commonly undergo short postharvest steps such as piling, drying, shelling, and temporary storage to complete post-ripening before oil extraction (Zhu et al., 2020; Yu et al., 2023; Zhu et al., 2024; Kong et al., 2025). These operations can reshape seed physiology and influence oil accumulation, fatty-acid composition, acid value, peroxide value, and the retention of nutritionally important minor compounds, indicating that the postharvest interval is a biologically active stage rather than a passive holding period (He et al., 2022; Yu et al., 2023; Zhu et al., 2024; Kong et al., 2025).

Among the lipid-soluble minor constituents of camellia oil, squalene is especially relevant because it is both a desirable bioactive quality trait and a central intermediate in plant sterol and triterpenoid biosynthesis (Li et al., 2022; Zhong et al., 2026). Recent work has shown that squalene in *C. oleifera* oil is sensitive to postharvest conditions: drying method affects its retention, and room-temperature storage reduces squalene more strongly than low-temperature storage (Kong et al., 2025). At the pathway level, squalene biosynthesis is primarily supplied by the mevalonate (MVA) pathway, and recent transcriptomic work in developing oil-tea seeds highlighted HMGR-associated MVA-pathway genes among the candidates linked to squalene accumulation (Gu et al., 2025; Kong et al., 2025). Therefore, clarifying how postharvest conditions reshape HMGR-associated responses is important not only for understanding seed metabolism but also for designing processing strategies that better preserve or enhance squalene-related quality traits (Gu et al., 2025; Kong et al., 2025).

Despite increasing interest in *C. oleifera* biology, most recent molecular studies have focused on drought tolerance, low-temperature responses during flowering, seed development, or metabolomic changes in fruits exposed to light and temperature during post-ripening (He et al., 2022; Wu et al., 2022, 2023; Ye et al., 2023), rather than on early HMGR-linked terpenoid responses in mature postharvest fruits. In parallel, postharvest studies have mainly emphasized drying, piling, storage, and conventional oil-quality indices (Zhu et al., 2020; Yu et al., 2023; Zhu et al., 2024; Kong et al., 2025). Together, these studies show that postharvest handling can alter seed physiology and oil quality, but the early molecular basis by which short-term postharvest warm conditioning affects squalene accumulation in seed-kernel tissues remains poorly resolved. It also remains unclear whether HMGR-associated MVA responses and phosphorylation-related signaling components are transcriptionally coordinated with the observed biochemical changes during this short industrially relevant interval (Wu et al., 2023; Gu et al., 2025; Kong et al., 2025). This unresolved interface between postharvest processing and kernel terpenoid metabolism constitutes the central knowledge gap addressed here (He et al., 2022).

In this study, we established a short postharvest warm conditioning regime using freshly harvested, fully mature *C. oleifera* fruits and collected seed-kernel tissues after treatment for integrative analysis. By combining RNA sequencing (RNA-seq), ultraperformance liquid chromatography–tandem mass spectrometry (UPLC-MS/MS) metabolomics, targeted squalene quantification, ELISA-based HMGR abundance measurements, and reverse-transcription quantitative PCR validation, we aimed to define the early molecular and metabolic signatures associated with warm conditioning-related squalene accumulation. We found that short-term warm conditioning coincided with increased squalene content and elevated HMGR abundance, together with broad transcriptomic reprogramming involving MVA-associated genes and multiple phosphorylation-related regulators, including mitogen-activated protein kinase (MAPK)-related and phosphatase-related components. Rather than claiming a fully resolved regulatory mechanism, this study provides an association-based framework linking an industrially relevant postharvest process to HMGR/MVA-associated responses and squalene accumulation in *C. oleifera* seed-kernel tissues.

## 2 Materials and methods

### 2.1 Plant material and growing area description

Fruits of *C. oleifera* cv. ‘Cenruan 3’ were collected from the experimental orchard of Guangxi Forestry Research Institute, No. 23 Yongwu Road, Xixiangtang District, Nanning, Guangxi Zhuang Autonomous Region, China (22°55′38″ N, 108°20′51″ E). The orchard is located in a humid subtropical monsoon region, with a mean annual temperature of approximately 21.8 °C and mean annual precipitation of about 1453.4 mm. The site belongs to the subtropical red-soil region of Guangxi, where red soils and lateritic red soils are the dominant regional soil types. The oil-tea trees used in this study were 7 years old at the time of sampling. The orchard was established in 2018 using grafted seedlings at a planting density of 70 trees per Chinese mu. Routine orchard management included precision drip irrigation according to crop water demand, basal fertilization combined with staged topdressing, nitrogen-oriented fertilization during the juvenile stage, and balanced N–P–K fertilization supplemented with micronutrients after the onset of full bearing; high-chloride fertilizers were avoided throughout cultivation. Pruning was carried out from postharvest until before spring shoot emergence to maintain an open-center or rounded canopy, remove diseased, weak, and crossing branches, and head back overly vigorous shoots to improve canopy aeration and light penetration. Fruits were harvested on November 1, 2025, shortly after Shuangjiang (the “Frost’s Descent” solar term in late autumn), when they had reached full maturity.

### 2.2 Treatment arrangement

Freshly harvested, fully mature *C. oleifera* fruits were assigned to three treatment groups: T-0 (untreated control), T-12, and T-24. For the postharvest warm conditioning treatment, intact fruits were incubated in darkness in an artificial climate chamber (RDN-1000C, 1000 L; Ningbo Yanghui Instrument Co., Ltd., Ningbo, Zhejiang, China) at 35 °C and 95% relative humidity for 12 or 24 h. Fruits in the T-0 group were collected immediately after harvest without warm conditioning. Each treatment comprised three biological replicates, and each replicate consisted of 200 fruits. After treatment, the pericarp and seed coat were removed, and the seed-kernel tissues were collected, immediately frozen in liquid nitrogen, and stored at −80 °C for subsequent transcriptomic and metabolomic analyses. Each biological replicate comprised pooled seed-kernel tissues obtained from 200 fruits (400 kernels per replicate).

### 2.3 Metabolite extraction

For metabolomic analysis, frozen seed-kernel samples were first lyophilized in a vacuum freeze dryer (Scientz-100F; Ningbo Scientz Biotechnology Co., Ltd., Ningbo, China) and then ground in a mixer mill (MM 400, Retsch GmbH, Haan, Germany) with a zirconia bead for 1.5 min at 30 Hz. A 50 mg aliquot of the powdered sample was extracted with 1.2 mL of 70% methanol and vortexed six times for 30 s at 30 min intervals. The mixture was then centrifuged at 8,050 × g for 3 min, and the supernatant was filtered through a 0.22 μm membrane filter (SCAA-104; ANPEL Laboratory Technologies, Shanghai, China) prior to UPLC-MS/MS analysis.

### 2.4 UPLC-MS/MS conditions

Sample extracts were analyzed on a UPLC–ESI–MS/MS platform consisting of a Nexera X2 UPLC system (Shimadzu, Kyoto, Japan) coupled to a QTRAP 4500 mass spectrometer (SCIEX, Framingham, MA, USA). Analytical conditions were as follows: Chromatographic separation was performed on an SB-C18 column (1.8 µm, 2.1 × 100 mm; Agilent Technologies, Santa Clara, CA, USA); mobile phase: solvent A (pure water with 0.1% formic acid) and solvent B (acetonitrile with 0.1% formic acid); gradient elution program: 95% A, 5% B; linear adjustment to 5% A, 95% B within 9 min and then hold for 1 min; adjustment to 95% A, 5.0% B within 1.1 min and then hold for 2.9 min; flow rate: 0.35 mL/min; column oven temperature: 40 °C; injection volume: 4 μL. The eluate was analyzed using an ESI-triple quadrupole-linear ion trap (QTRAP)-MS system.

### 2.5 ESI-QTRAP-MS/MS

The ESI source operation parameters were as follows: source temperature: 550 °C; ion spray voltage: 5,500 V (positive ion mode)/−4,500 V (negative ion mode); ion source gas I, gas II, and curtain gas: 50, 60, and 25 psi, respectively; collision-activated dissociation: high. Instrument tuning and mass calibration were performed using 10 and 100 μmol/L polypropylene glycol solutions in QQQ and LIT modes, respectively. QQQ scans were acquired in MRM experiments with the collision gas (nitrogen) set to medium. Declustering potential and collision energy were optimized for individual MRM transitions. A specific set of MRM transitions was monitored for each period according to the metabolites eluted within that period.

### 2.6 Qualitative and quantitative metabolite analyses

Metabolite data were log_2_-transformed and normalized before statistical analysis. An unsupervised principal component analysis (PCA) was performed using the prcomp function in R (www.r-project.org). Data were unit-variance scaled before PCA. A hierarchical cluster analysis (HCA) of samples and metabolites was performed and the results were visualized in heatmaps with dendrograms. Pearson correlation coefficients (PCC) were calculated using the cor function in R and presented in heatmaps. HCA was completed and PCC was calculated using the R package ComplexHeatmap. For HCA, normalized signal intensities of metabolites (after unit variance scaling) were visualized as a color spectrum. For a two-group analysis, differentially accumulated metabolites were determined using the following criteria: VIP ≥ 1 and |log_2_(fold-change)| ≥ 1.0. VIP values were extracted from OPLS-DA data, which were presented in score plots and permutation plots generated using the R package MetaboAnalystR. Data were log-transformed and mean-centered before performing OPLS-DA. To avoid overfitting, a permutation test (200 permutations) was completed. Identified metabolites were annotated using the KEGG Compound database (http://www.kegg.jp/kegg/compound/), after which annotated metabolites were mapped using the KEGG Pathway database (http://www.kegg.jp/kegg/pathway.html). Pathways with significantly regulated metabolites were selected for a metabolite set enrichment analysis using the MSEA online server; their significance was determined on the basis of hypergeometric test p-values. To ensure annotation accuracy, all metabolite identifications were manually validated by matching retention times, precursor ions (m/z), and MS/MS fragmentation spectra with authentic standards in the MetWare MWDB database. KEGG assignments were curated to retain only plant-related metabolic pathways prior to visualization.

### 2.7 RNA extraction, RNA-seq analysis, and RT–qPCR

Total RNA was extracted using the Total RNA Extractor (Trizol) kit (Sangon, China) and then treated with RNase-free DNase I to remove any remaining genomic DNA. RNA integrity was evaluated by 1.0% agarose gel electrophoresis, whereas RNA quality and quantity were determined using a NanoPhotometer^®^ spectrophotometer (IMPLEN, CA, USA) and an Agilent 2100 Bioanalyzer (Agilent Technologies, CA, USA). High-quality RNA samples were used for library construction and sequencing by Sangon Biotech (Shanghai) Co., Ltd. For each sample, 2 μg RNA was used as the input material for constructing sequencing libraries using a VAHTSTM mRNA-seq v2 Library Prep Kit for Illumina^®^. Index codes were added to attribute sequences to each sample. Briefly, mRNA was purified from total RNA using poly-T oligo-attached magnetic beads and then fragmented using divalent cations at high temperatures in VAHTS™ First Strand Synthesis Reaction Buffer (5×). First-strand cDNA was synthesized using a random hexamer primer and M-MuLV Reverse Transcriptase (RNase H-). The second cDNA strand was synthesized using DNA polymerase I and RNase H. The remaining overhangs were converted to blunt ends via exonuclease/polymerase activities. After the 3′ ends of cDNA fragments were adenylated, an adapter was ligated to the fragments. An AMPure XP system (Beckman Coulter, Beverly, USA) was used to select cDNA fragments with the preferred length (150–200 bp), after which 3 μL USER Enzyme (NEB, USA) was added to the size-selected cDNA. The mixture was incubated at 37 °C for 15 min and then at 95 °C for 5 min. A PCR amplification was performed using Phusion High-Fidelity DNA polymerase, Universal PCR primers, and an Index (X) Primer. PCR products were purified (AMPure XP system) and library quality was assessed using the Agilent Bioanalyzer 2100 system. High-quality libraries were then quantified and pooled for the paired-end sequencing performed on a HiSeq XTen system (Illumina, San Diego, CA).

RT–qPCR was performed to validate the RNA-seq results. Total RNA was extracted, quality assessed, and reverse-transcribed from 1,500 ng per sample into cDNA (65 °C for 5 min; 25 °C for 10 min, 50 °C for 30 min, and 85 °C for 5 min). cDNA was stored at −20 °C, diluted 10-fold, and amplified on an ABI 7500 using 2× SGExcel FastSYBR Mixture (20 μL: 10 μL 2× mix, 0.4 μL each primer at 10 μM, 2 μL diluted cDNA, 7.2 μL nuclease-free water) under the following program: 95 °C for 3 min; 45 cycles of 95 °C for 15 s and 60 °C for 45 s; melt-curve analysis. Relative expression was normalized to SAND and calculated by the 2^−ΔΔCt method, with technical replicates for all samples.

### 2.8 Data assessment and quality control

Raw reads were assessed using FastQC (v0.11.2). Adapter sequences and low-quality bases were removed with Trimmomatic (v0.36) using standard filters: (1) adapter trimming; (2) removal of leading and trailing bases with Phred quality < 20; (3) sliding-window trimming (window size 5 bp) requiring an average Phred quality ≥ 20; and (4) removal of reads shorter than 35 nt after trimming, together with their paired mates. The resulting clean reads were used for downstream analyses.

### 2.9 Transcriptome assembly and gene annotation

Clean reads were *de novo* assembled into transcripts using Trinity (version 2.0.6) (parameter: min_kmer_cov 2). Transcripts with a minimum length of 200 bp were clustered to minimize redundancy. For each cluster (representing the transcriptional complexity for the same gene), the longest sequence was preserved and designated as a unigene. Unigenes served as queries for a BLAST search of the following databases: NCBI Nr (non-redundant protein database), Swiss-Prot, TrEMBL, CDD (Conserved Domain Database), Pfam, and KOG (EuKaryotic Orthologous Groups) (E-value < 1e-5). The best alignments were used to determine unigene open reading frames and the encoded amino acid sequences. TransDecoder (version 3.0.1) was used to predict the coding sequences of the unaligned unigenes. Gene Ontology (GO) functional annotation information was obtained for the transcripts annotated by Swiss-Prot and TrEMBL. KAAS (KEGG Automatic Annotation Server version 2.1) was used for Kyoto Encyclopedia of Genes and Genomes (KEGG) annotations.

### 2.10 RNA-seq and expression analysis

Bowtie2 (version 2.3.2) was used to map quality-controlled reads to the assembled transcripts, and RSeQC (version 2.6.1) was used to summarize the alignment statistics. Salmon (version 0.8.2) was used to calculate read counts and unigene expression values. TPM (transcripts per million) corrects for gene length and sequencing depth, enabling direct comparison of expression levels across samples. Principal component analysis (PCA) and principal coordinates analysis (PCoA) were performed to reflect the distance and difference between samples. DESeq2 (version 1.12.4) was used to determine differentially expressed genes (DEGs) between two samples. Genes were considered significantly differentially expressed if q-value < 0.05 and |fold change| > 2. When the normalized expression of a gene was zero between two samples, its expression value was adjusted to 0.01 (as 0 cannot be plotted on a log plot). If the normalized expression of a certain gene in two libraries was all lower than 1, further differential expression analysis was conducted without this gene.

### 2.11 Functional characterization of DEGs

GO and KEGG analyses were performed to functionally characterize DEGs. The GO database is part of an international standard classification system for gene functions. DEGs were annotated with GO terms (biological functions). The number of genes annotated with each term was recorded. A hypergeometric test was conducted to identify significantly enriched GO terms in the gene list. The KEGG database is a public database of pathways. A KEGG pathway analysis involving a hypergeometric test was completed to reveal significantly enriched metabolic pathways or signal transduction pathways among DEGs. A false discovery rate (q-value) < 0.05 was used as the threshold for determining the significance of GO terms and KEGG pathways.

### 2.12 HMGR ELISA-based abundance measurement and squalene quantification

HMGR abundance was measured using a commercial plant HMGR ELISA kit based on a double-antibody sandwich format, according to the manufacturer’s instructions. Crude extracts were prepared from *C. oleifera* seed-kernel tissues on ice, clarified by centrifugation, and loaded into 96-well plates together with blanks and serially diluted standards. Samples were added as 40 μL diluent plus 10 μL extract (final dilution, 5-fold), color was developed with TMB, and absorbance was read at 450 nm. The assay was calibrated using six standards spanning 0–80 U/L. Linear regression of OD450 against the kit-defined standard values yielded the equation y = 47.8619x − 2.7083 (R² = 0.9975), where x represents OD450 and y represents the kit-defined HMGR abundance output. HMGR abundance values were calculated from this calibration curve, corrected by the total dilution factor, and, where indicated, converted to U/g FW using extraction volume and tissue mass. For the target seed-kernel samples analyzed in this study, the T-12 and T-24 groups fell within the calibration range, whereas the T-0 group was slightly above the upper calibrator. Assay reproducibility was evaluated from replicate wells, with within-assay coefficients of variation of 2.0%, 5.3%, and 2.4% for the T-0, T-12, and T-24 seed-kernel samples, respectively.

Squalene was quantified by GC-MS using a TRACE GC 1300 gas chromatograph coupled to an ISQ LT mass spectrometer (Thermo Fisher Scientific) equipped with a TG-5MS capillary column (30 m × 0.25 mm i.d., 0.25 μm film thickness). Finely ground seed-kernel tissues were extracted with dichloromethane (DCM) by ultrasonication (5 mL, 30 min), followed by centrifugation; the residue was re-extracted with DCM (5 mL, 10 min). The two supernatants were combined and brought to a final volume of 10 mL with DCM before analysis. GC conditions were as follows: helium carrier gas at 0.8 mL min^-1^, split ratio 40:1, an injection volume of 2 μL, and an injector temperature of 300 °C. The oven temperature program was held at 80 °C for 2 min, ramped at 20 °C min^-1^ to 300 °C, and held for 15 min. MS conditions were electron impact (EI) ionization at 70 eV, with an ion source temperature of 220 °C and a transfer line temperature of 280 °C. Data were acquired in selected-ion monitoring (SIM) mode using m/z 81, 217, 368, and 386. Quantification was performed using an external calibration curve prepared from authentic squalene standards. Squalene contents were expressed as μg/g FW of seed-kernel tissues.

### 2.13 Statistical analysis

All experiments were conducted with three biological replicates unless otherwise stated. Data are presented as mean ± standard deviation (SD). Differences among treatments (T-0, T-12, and T-24) were evaluated by one-way analysis of variance (ANOVA) followed by Tukey’s honestly significant difference (HSD) test for multiple comparisons. When data did not meet the assumptions of normality or homogeneity of variance, the Kruskal–Wallis nonparametric test was applied. Statistical significance was defined as *P* < 0.05. All analyses were performed using SPSS Statistics 26.0 (IBM, Armonk, NY, USA). In addition to fold changes, effect sizes were calculated for the principal biochemical and transcriptional comparisons. For pairwise comparisons against T-0, *Hedges’ g* was used to account for the small sample size.

## 3 Results and discussion

### 3.1 Phenotypic and physiological indices

**Figure 1:**
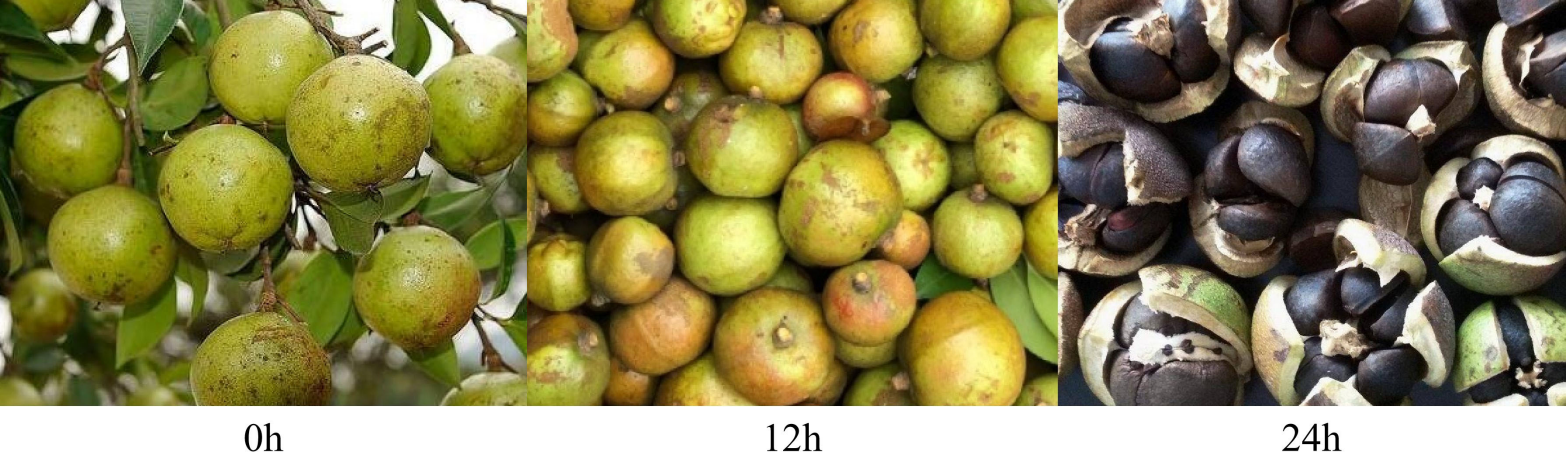
Representative appearance of intact *C. oleifera* fruits after incubation at 35 °C for 0, 12, and 24 h. Biochemical and multi-omics analyses were performed using seed-kernel tissues dissected from treated fruits (n = 3 biological replicates per time point).

The effects of postharvest warm conditioning on freshly harvested *C. oleifera* fruits were first assessed by phenotypic observations and associated biochemical measurements. After 24 h of treatment, fruits exhibited obvious pericarp cracking (**Figure 1**). Consistent with the quantitative measurements summarized in **Table 1**, squalene content in seed-kernel tissues increased significantly after 12 h and remained significantly elevated after 24 h of treatment. Specifically, squalene content increased from 177.58 ± 8.2 μg/g in T-0 to 314.30 ± 9.7 μg/g in T-12 and 309.10 ± 7.5 μg/g in T-24, corresponding to approximately 1.77-fold and 1.74-fold of the control, respectively. Based on these phenotypic and biochemical changes, samples from the three time points (0, 12, and 24 h) were selected for subsequent transcriptomic and metabolomic profiling.

**Table 1:** Quantitative changes in squalene content and HMGR abundance in *C. oleifera* seed-kernel tissues during postharvest warm conditioning.

Treatment	Squalene (μg/g)	HMGR abundance (U/g FW)	Effect size vs T-0 (squalene)	Effect size vs T-0 (HMGR abundance)
T-0	177.58 ± 8.2	10.70 ± 0.52	–	–
T-12	314.30 ± 9.7*	13.74 ± 0.48*	12.18	4.86
T-24	309.10 ± 7.5*	14.03 ± 0.55*	13.39	4.98

Values are presented as mean ± SD of three biological replicates. Asterisks (*) indicate significant differences relative to T-0 at *P* < 0.05. Effect sizes were calculated as *Hedges’ g* for pairwise comparisons against T-0.

### 3.2 Global transcriptomic profiles and expressed-gene overview during postharvest warm conditioning

**Figure 2:**
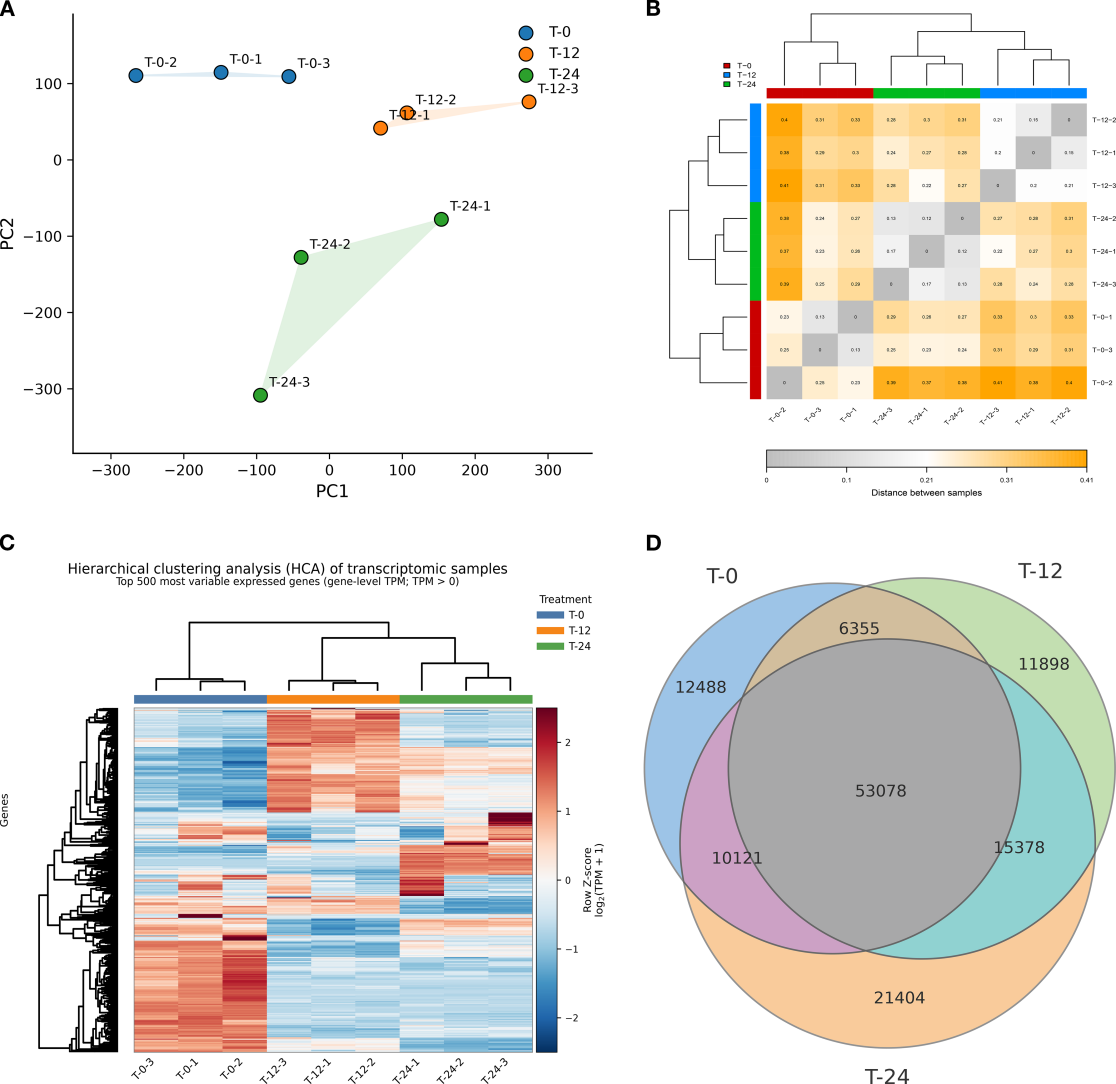
Global transcriptomic profiles and expressed-gene overview of *C. oleifera* seed-kernel tissues during postharvest warm conditioning. **(A)** Principal component analysis (PCA) of transcriptomic samples collected at T-0, T-12, and T-24. Each point represents one biological replicate. **(B)** Sample-to-sample distance heatmap showing the overall relationships among transcriptomic samples. **(C)** Hierarchical clustering analysis (HCA) heatmap based on the top 500 most variable expressed genes across all transcriptomic samples. Values are shown as row-scaled log_2_(TPM + 1). **(D)** Venn diagram showing shared and treatment-specific expressed genes across T-0, T-12, and T-24. Expressed genes were defined as transcripts with TPM > 0 within each treatment group.

To characterize transcriptomic changes during postharvest warm conditioning, freshly harvested *C. oleifera* fruits were incubated at 35 °C for 0, 12, and 24 h. After treatment, the pericarp and seed coat were removed, and seed-kernel tissues were collected for RNA sequencing (RNA-seq). After filtering low-quality reads, 62.38 Gb of clean data were obtained. The Q30 values exceeded 92.85%, and the GC content ranged from 50.12% to 54.48%, indicating high sequencing quality. In total, 131,511 assembled transcripts were annotated. We first assessed the global transcriptomic profiles using complementary sample-level analyses. Principal component analysis (PCA) showed clear separation among the T-0, T-12, and T-24 groups, whereas biological replicates clustered closely within each treatment, indicating good within-group reproducibility together with marked treatment-associated transcriptomic divergence (**Figure 2A**). A sample-to-sample distance heatmap further supported this pattern by showing shorter distances among replicates from the same treatment and larger distances between treatments (**Figure 2B**). Consistently, hierarchical clustering analysis (HCA) based on the top 500 most variable expressed genes resolved the samples into treatment-dependent clusters, with T-24 clearly separated from T-0 and T-12 occupying an intermediate but distinct position (**Figure 2C**). Together, these analyses indicate progressive global transcriptomic divergence during warm conditioning. To complement this sample-level overview, we also summarized expressed-gene overlap across treatments. The majority of expressed genes were shared among T-0, T-12, and T-24, whereas each treatment also retained a distinct subset of treatment-specific expressed genes (**Figure 2D**). This pattern suggests that postharvest warm conditioning operated on a broadly shared transcriptional background while being accompanied by time-dependent shifts in the expressed-gene repertoire. Overall, these results support clear and stage-associated global transcriptomic reprogramming in *C. oleifera* seed-kernel tissues during postharvest warm conditioning.

### 3.3 Differential transcriptomic responses during postharvest warm conditioning

**Figure 3:**
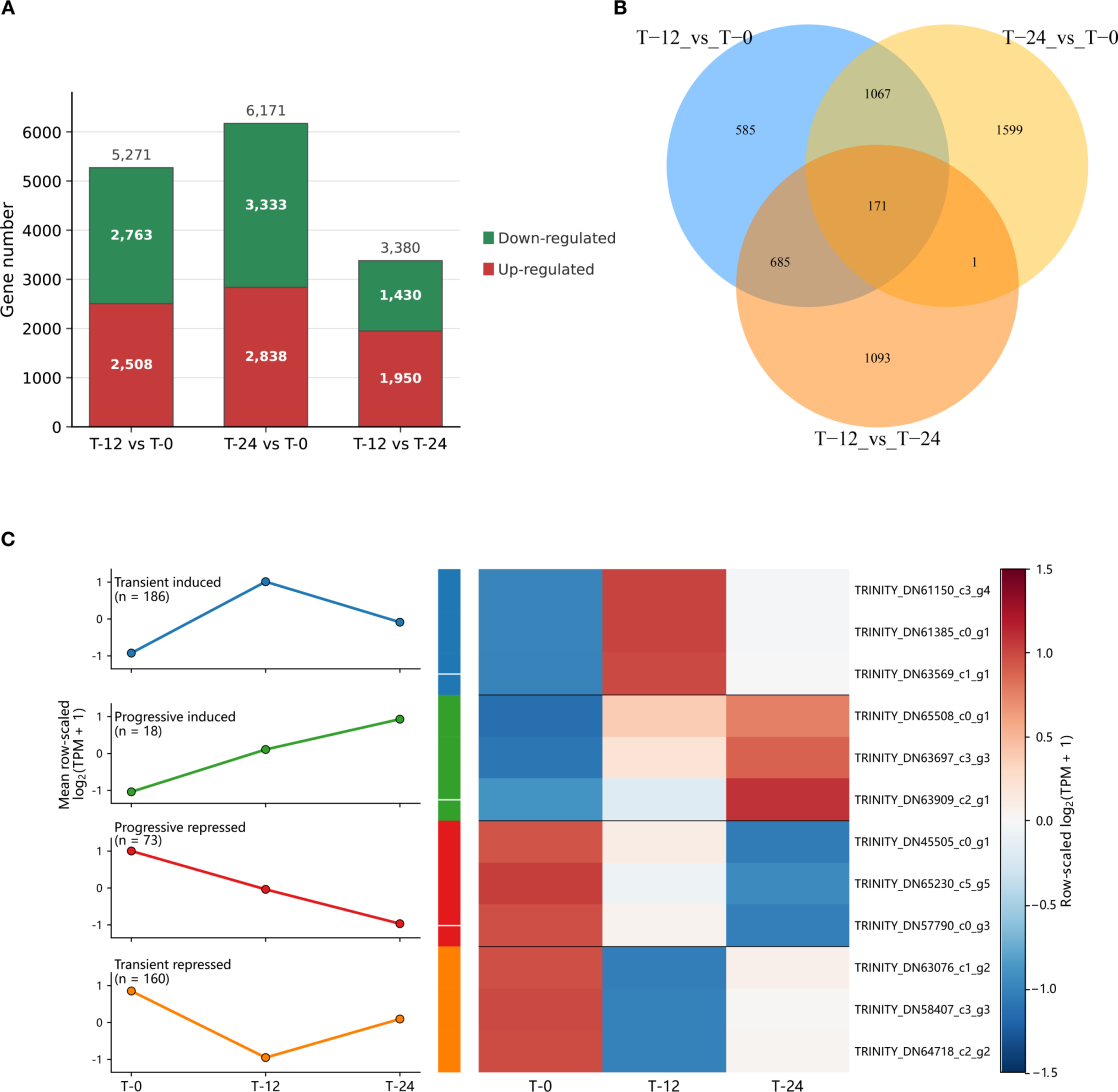
Differential transcriptomic responses in *C. oleifera* seed-kernel tissues during postharvest warm conditioning. **(A)** Numbers of upregulated and downregulated differentially expressed genes (DEGs) identified in the three pairwise comparisons: T-12 vs T-0, T-24 vs T-0, and T-12 vs T-24. DEGs were defined using a q-value < 0.05 and an absolute fold change > 2. **(B)** Venn diagram showing the overlap of DEGs among the three pairwise comparisons. **(C)** Representative temporal expression modules derived from treatment-responsive genes. The line plots on the left show the mean row-scaled log_2_(TPM + 1) trajectories for four major response patterns: transient induced, progressive induced, progressive repressed, and transient repressed. Numbers in parentheses indicate the number of genes assigned to each module. The heatmap on the right shows representative genes from each module across T-0, T-12, and T-24. Color intensity represents row-scaled log_2_(TPM + 1) abundance. Volcano plots for the three pairwise comparisons are provided in **Supplementary Figures S1A–C**.

To further define comparison-specific transcriptomic responses, we next examined differential gene expression across the three pairwise comparisons. Differentially expressed genes (DEGs) were identified using a q-value < 0.05 and an absolute fold change > 2. In total, 5,271 DEGs were detected in T-12 vs T-0, including 2,508 upregulated and 2,763 downregulated genes. The T-24 vs T-0 comparison yielded 6,171 DEGs, comprising 2,838 upregulated and 3,333 downregulated genes, whereas T-12 vs T-24 yielded 3,380 DEGs, including 1,950 upregulated and 1,430 downregulated genes (**Figure 3A**). Volcano plots for the three pairwise comparisons are provided in **Supplementary Figures S1A–C** and are consistent with the DEG distributions summarized in **Figure 3A**. To complement the comparison-specific results, we next examined the overlap among DEG sets. A total of 171 DEGs were shared across all three pairwise comparisons, whereas the remaining DEG subsets were either comparison-specific or shared between only two contrasts (**Figure 3B**). This shared DEG fraction points to a core transcriptomic response associated with postharvest warm conditioning, while the non-overlapping fractions are more consistent with stage-dependent transcriptional adjustments.

To move beyond overlap counts alone, we further examined the temporal behavior of treatment-responsive genes. Representative clustering identified four major dynamic expression modules: transient induced (n = 186), progressive induced (n = 18), progressive repressed (n = 73), and transient repressed (n = 160) (**Figure 3C**). Genes in the transient-induced module showed marked upregulation at 12 h followed by a decline toward baseline at 24 h, whereas genes in the progressive-induced module increased continuously from T-0 to T-24. In contrast, genes in the progressive-repressed module declined monotonically across the time course, while those in the transient-repressed module showed early suppression at 12 h followed by partial recovery at 24 h. The accompanying heatmap further highlights the contrasting temporal trajectories of representative genes across T-0, T-12, and T-24 (**Figure 3C**). Together, these results indicate that postharvest warm conditioning triggered not only extensive differential gene expression but also structured temporal reprogramming in *C. oleifera* seed-kernel tissues. The combination of comparison-specific DEG abundance, a shared core DEG set, and distinct temporal expression patterns provides a basis for subsequent pathway-enrichment analysis and downstream biological interpretation.

### 3.4 Functional annotation, pathway enrichment, and a candidate association framework of postharvest warm conditioning responses

**Figure 4:**
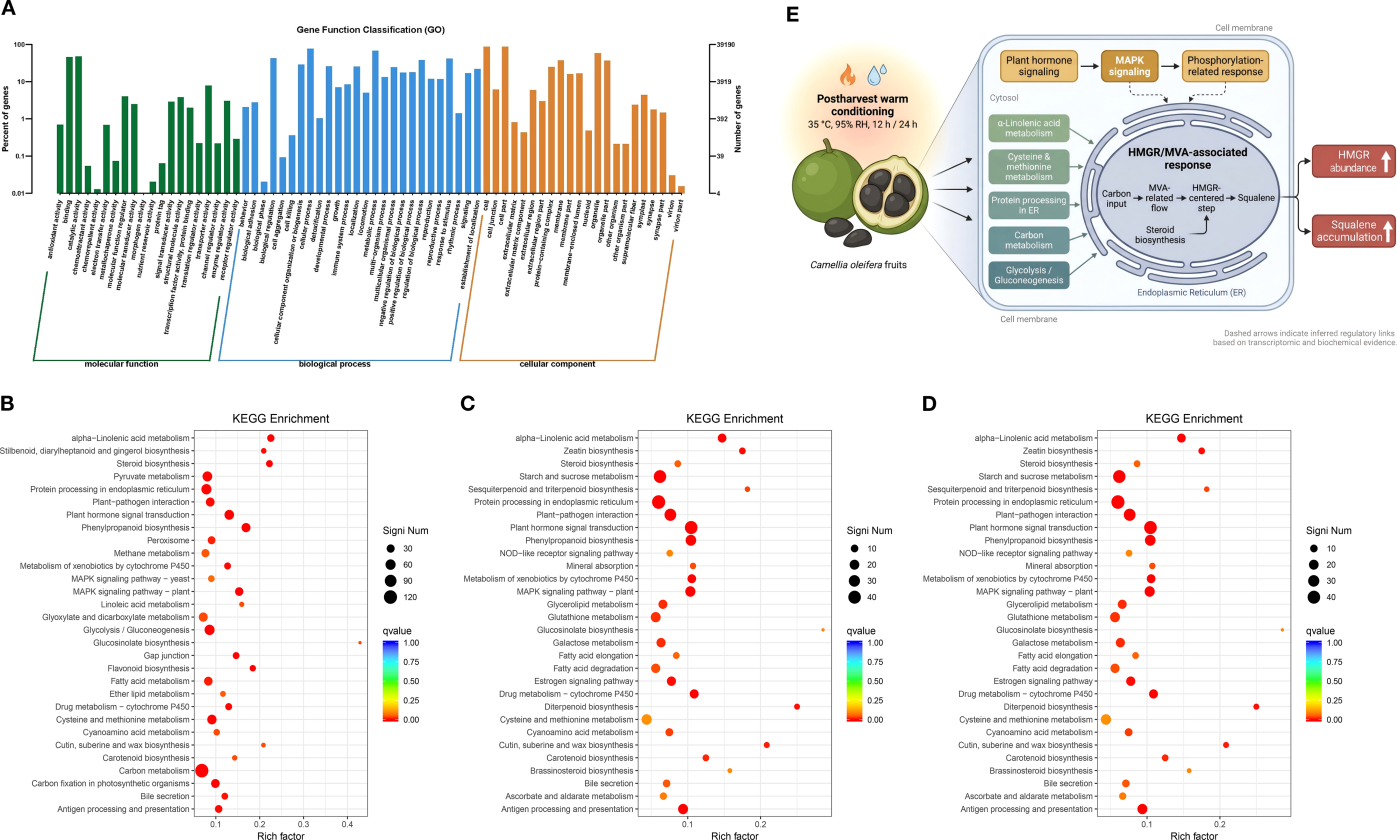
Functional annotation, KEGG pathway enrichment, and a candidate association framework of postharvest warm conditioning responses in *C. oleifera* seed-kernel tissues. **(A)** Gene Ontology (GO) classification of all annotated genes at level 2, grouped into biological process, cellular component, and molecular function. The left y-axis shows the percentage of genes assigned to each term, and the right y-axis shows the corresponding gene counts. **(B–D)** KEGG enrichment bubble plots of differentially expressed genes (DEGs) for T-12 vs T-0 **(B)**, T-12 vs T-24 **(C)**, and T-24 vs T-0 **(D)**. The x-axis indicates the rich factor. Bubble size represents DEG number (SigniNum), and bubble color indicates enrichment significance (q-value). **(E)** Candidate association framework linking postharvest warm conditioning with HMGR/MVA-associated responses and squalene accumulation in *C. oleifera* seed-kernel tissues. The framework integrates the major transcriptomic features identified in this study, including hormone- and MAPK-related signaling, phosphorylation-associated responses, membrane and protein remodeling, and carbon metabolic support, together with increased HMGR abundance and squalene accumulation.

To place the postharvest warm conditioning response in a broader functional context, all annotated genes were first assigned to Gene Ontology (GO) categories encompassing biological process, cellular component, and molecular function (**Figure 4A**). Within biological process, genes were predominantly associated with response to stimulus, biological regulation, metabolic process, cellular process, and cellular component organization or biogenesis, indicating that the transcriptome encompassed broad stimulus-responsive and metabolic functions. In the cellular component category, organelle, membrane, cell part, and organelle part were among the most represented terms, highlighting strong representation of membrane- and organelle-associated functions. In molecular function, catalytic activity and binding predominated, suggesting that many annotated genes encoded enzymes and proteins with molecular interaction capacity. Together, these GO assignments provided a coherent functional backdrop for interpreting the warm-conditioning-responsive transcriptome in *C. oleifera* seed-kernel tissues.

We next examined pathway-level responses using Kyoto Encyclopedia of Genes and Genomes (KEGG) enrichment analysis of differentially expressed genes (DEGs) across the three pairwise comparisons (**Figures 4B–D**). Despite comparison-specific differences, the enriched pathways converged on three closely connected functional layers. First, stress- and signaling-related pathways were repeatedly over-represented, most notably plant hormone signal transduction and MAPK signaling pathway–plant, indicating that postharvest warm conditioning elicited broad transcriptional engagement of canonical stress-signaling modules (Chen et al., 2024; Zheng et al., 2024). Second, pathways associated with protein and cellular homeostasis were prominently enriched, including protein processing in the endoplasmic reticulum and, particularly at later stages, information-processing and proteostasis-related pathways such as ribosome, spliceosome, and proteasome (Jo et al., 2025). Third, central carbon metabolism and lipid-associated pathways were consistently represented, including starch and sucrose metabolism, glycolysis/gluconeogenesis, pyruvate/carbon metabolism, and lipid-related terms such as α-linolenic acid metabolism, linoleic acid metabolism, and fatty acid metabolism. These enrichment profiles indicate coordinated transcriptional reprogramming involving stress signaling, proteostasis, and remodeling of carbon and lipid metabolism during postharvest warm conditioning.

To integrate pathway-level signatures with the observed biochemical phenotype, we established a candidate association framework that summarizes the principal relationships supported by the present dataset (**Figure 4E**). Within this framework, postharvest warm conditioning is linked to hormone- and MAPK-related signaling, phosphorylation-related responses, membrane and protein remodeling, and carbon metabolic support, alongside increased HMGR abundance and squalene accumulation. The schematic offers a consolidated view of the transcriptomic and biochemical response landscape revealed in this study.

### 3.5 Dynamic analysis of transcriptome data

**Figure 5:**
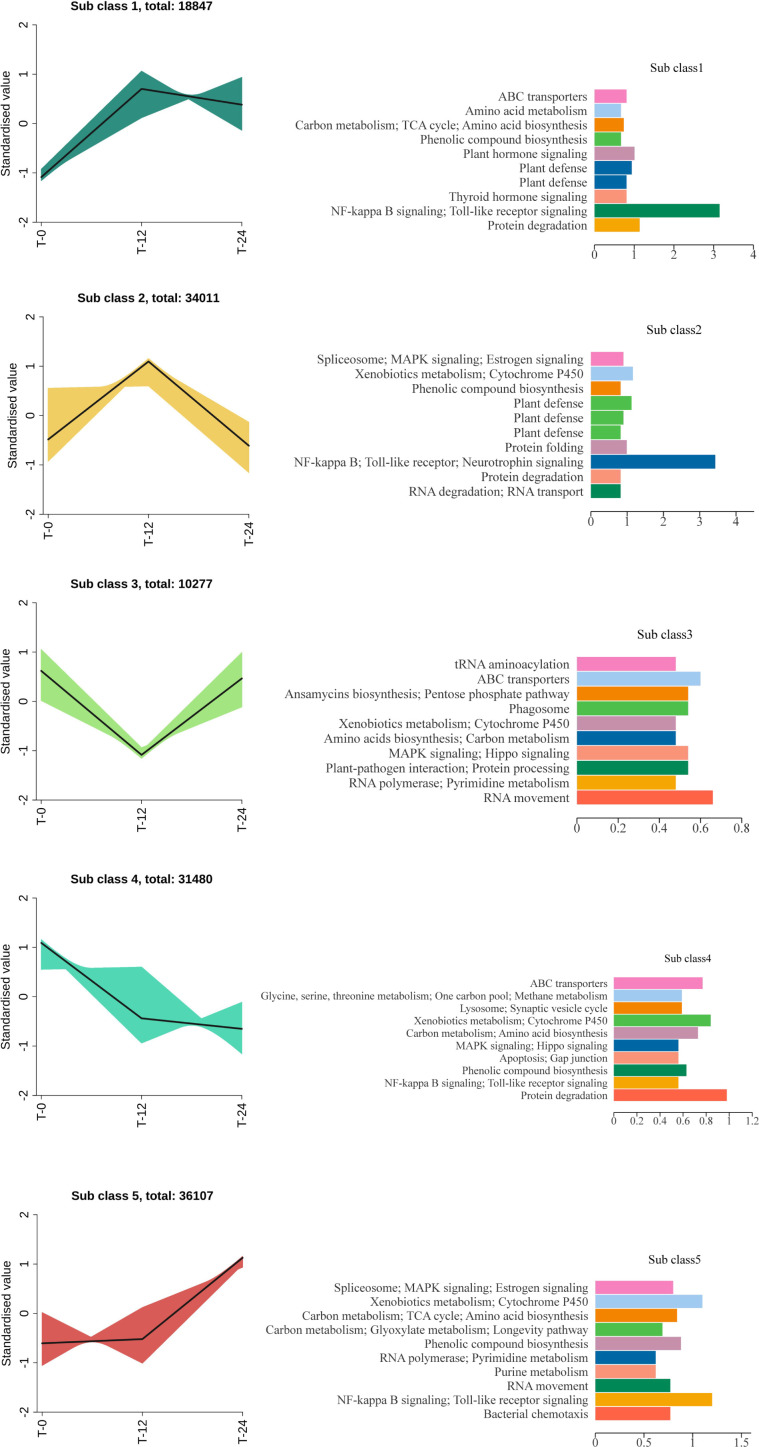
K-means transcriptomic analysis of the effects of postharvest warm conditioning. DEGs are divided into five subclasses. The top 10 KEGG pathways in each subclass are listed.

Based on K-means clustering analysis (**Figure 5**), gene expression patterns in harvested *C. oleifera* seed-kernel tissues during postharvest warm conditioning were partitioned into five subclasses. Genes in each subclass had different expression patterns at different treatment time points (T-0, T-12, and T-24) and were associated with different KEGG pathways. Analyses of gene expression patterns in different subclasses revealed significant spatiotemporal changes, reflecting the multiple biological responses of *C. oleifera* seed-kernel tissues to postharvest warm conditioning.

The expression level of subclass 1 genes stabilized after an initial rapid increase in the early stage of the postharvest warm conditioning (T-12); the enriched KEGG pathways among these genes were mainly carbon metabolism, TCA cycle, amino acid biosynthesis, phenolic compound synthesis, and plant hormone signaling. Considering their expression trends, the subclass 1 genes were rapidly activated during the early response to postharvest warm conditioning, which helped maintain basic cellular metabolism and stress defense responses. Subclass 2 gene expression levels decreased rapidly after peaking at T-12; the enriched KEGG pathways were mainly related to stress responses, including MAPK signaling pathway, endoplasmic reticulum protein folding, protein degradation, and RNA splicing. These genes showed a typical stress-induced expression pattern and may encode key regulators of early stress-responsive signaling memory, post-transcriptional modifications, and cellular homeostasis. Enrichment of MAPK signaling in subclass 2 highlights the engagement of stress-responsive signaling programs during postharvest warm conditioning and situates this cluster within the broader adaptive response landscape. Subclass 3 gene expression levels were lowest at T-12, but subsequently increased; the enriched KEGG pathways among these genes included glycolysis/gluconeogenesis, glutathione metabolism, ABC transporters, and PI3K–Akt signaling pathway. This suggests that These genes may contribute to transient suppression of energy metabolism during the middle phase of postharvest warm conditioning, followed by metabolic recovery and activation of antioxidant and transporter functions. Hence, these genes may encode proteins with important roles for the postharvest warm conditioning recovery stage. The expression of subclass 4 genes continued to decrease during the treatment period; the enriched KEGG pathways among these genes included lipid metabolism, amino acid metabolism, ubiquitin-mediated protein degradation, and Toll/NF-κB signaling. Their expression trends and enriched KEGG pathways indicated that subclass 4 genes may be related to non-critical growth and development, which are negatively regulated during postharvest warm conditioning to decrease energy consumption. Moreover, they may be associated with programmed cell death and aging processes. Subclass 5 gene expression levels were most significantly upregulated at T-24; the main enriched KEGG pathways among these genes included RNA splicing, carbon metabolism, TCA cycle, phenylpropanoid synthesis, and two-component systems. The expression levels of these genes were consistent with a typical “late repair” response trend. These genes may contribute to cellular reconstruction, metabolic recovery, and physiological homeostasis-related regulation during the late stage of the postharvest warm conditioning response.

In summary, the K-means dynamic clustering analysis revealed the heterogeneity of the transcription-level responses of *C. oleifera* seed-kernel tissues during postharvest warm conditioning conditions. Various genes were associated with key time-specific KEGG pathways, including energy metabolism, signal transduction, protein processing, and transport, reflecting a multi-stage regulatory pattern in *C. oleifera* seed-kernel tissues, spanning early response, regulatory buffering, and subsequent adaptive recovery during postharvest warm conditioning. Notably, signaling-related genes in subclasses 1 and 5 exhibited temporal profiles broadly aligned with those of HMGR transcripts, pointing to coordinated transcriptional dynamics within the warm conditioning response.

### 3.6 Global metabolomic landscape during postharvest warm conditioning

**Figure 6:**
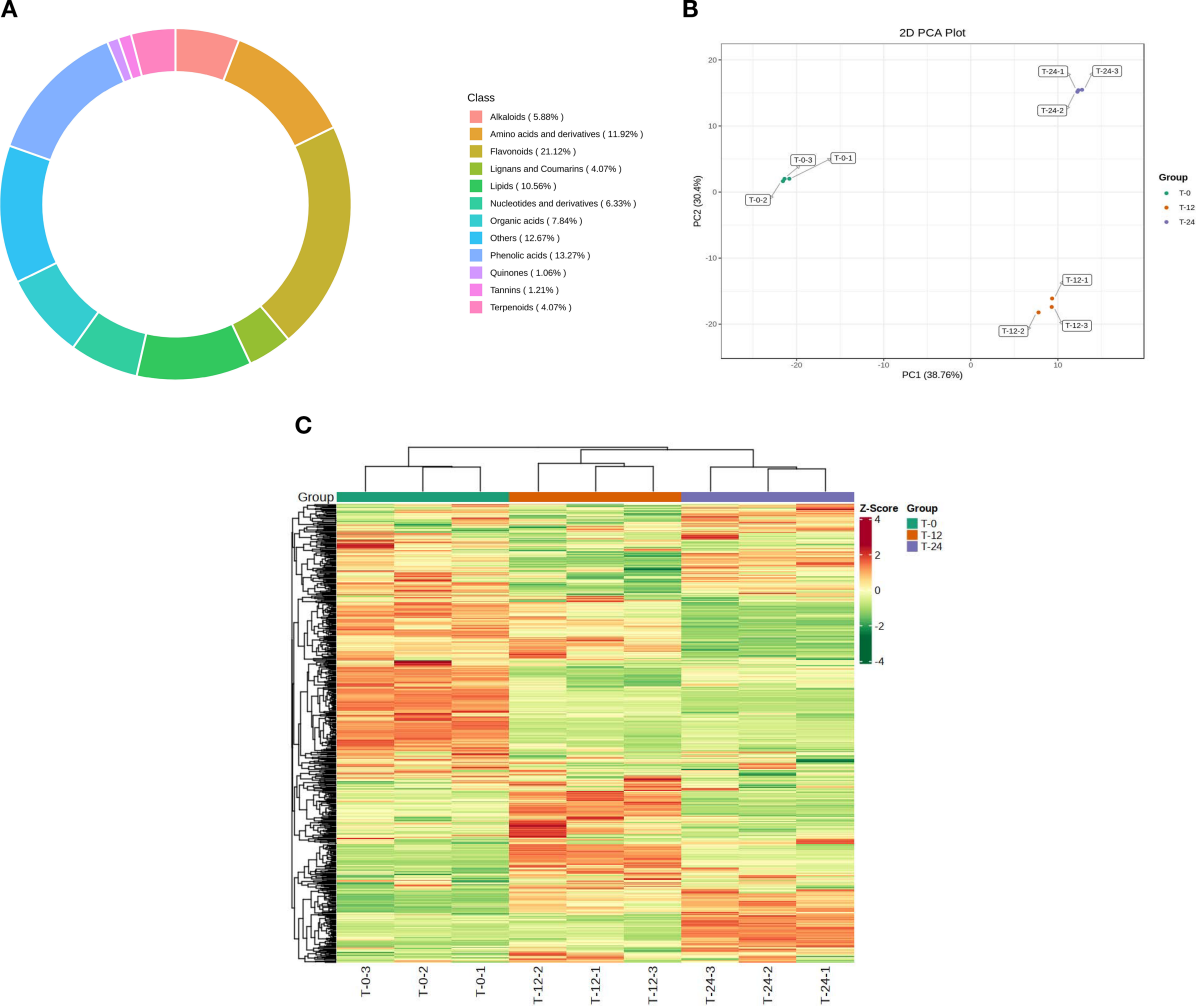
Global metabolomic profiles of *C. oleifera* seed-kernel tissues during postharvest warm conditioning. **(A)** Relative composition of metabolite classes among the 663 detected metabolites identified by UPLC-MS/MS. **(B)** Principal component analysis (PCA) score plot based on all detected metabolites from T-0, T-12, and T-24 samples. Each point represents one biological replicate. **(C)** Hierarchical clustering heatmap based on all detected metabolites across the nine samples. Columns represent samples, rows represent metabolites, and metabolite abundances are shown as Z-score–scaled relative levels. The top color bar indicates sample grouping.

To establish a global view of metabolic variation during postharvest warm conditioning, seed-kernel samples collected at T-0, T-12, and T-24 were subjected to widely targeted metabolomic profiling using a UPLC-MS/MS platform. In total, 663 metabolites were detected across the nine samples, indicating broad coverage of the seed-kernel metabolome. The annotated metabolite set was dominated by flavonoids (21.12%), phenolic acids (13.27%), other metabolites (12.67%), amino acids and derivatives (11.92%), and lipids (10.56%), with additional contributions from organic acids, nucleotides and derivatives, alkaloids, lignans and coumarins, terpenoids, tannins, and quinones (**Figure 6A**). This class distribution indicates that the *C. oleifera* seed-kernel metabolome under the present experimental conditions comprises a chemically diverse mixture of primary and secondary metabolites.

We next assessed the overall metabolomic relationships among samples using principal component analysis (PCA). The PCA score plot based on all detected metabolites clearly separated the three treatment groups, with PC1 explaining 38.76% of the variance and PC2 explaining 30.40% (**Figure 6B**). Biological replicates clustered tightly within each group, indicating good reproducibility of the metabolomic dataset. The T-12 and T-24 samples were both clearly separated from T-0, while remaining distinguishable from one another, supporting a strong and time-dependent shift in the metabolic state of seed-kernel tissues during postharvest warm conditioning.

Hierarchical clustering analysis further supported this global pattern. The heatmap based on all detected metabolites showed clear sample grouping by treatment, with replicates from the same time point clustering together and conditioned samples showing broad abundance shifts relative to the T-0 control (**Figure 6C**). Notably, the T-12 and T-24 groups showed related but distinct clustering structure, suggesting that substantial metabolic reorganization was already evident by 12 h and became more pronounced by 24 h. Together, these results show that postharvest warm conditioning induced pronounced and temporally structured remodeling of the *C. oleifera* seed-kernel metabolome, thereby providing the global metabolic context for subsequent analysis of differentially accumulated metabolites.

### 3.7 Differential metabolite accumulation and shared response patterns during postharvest warm conditioning

**Figure 7:**
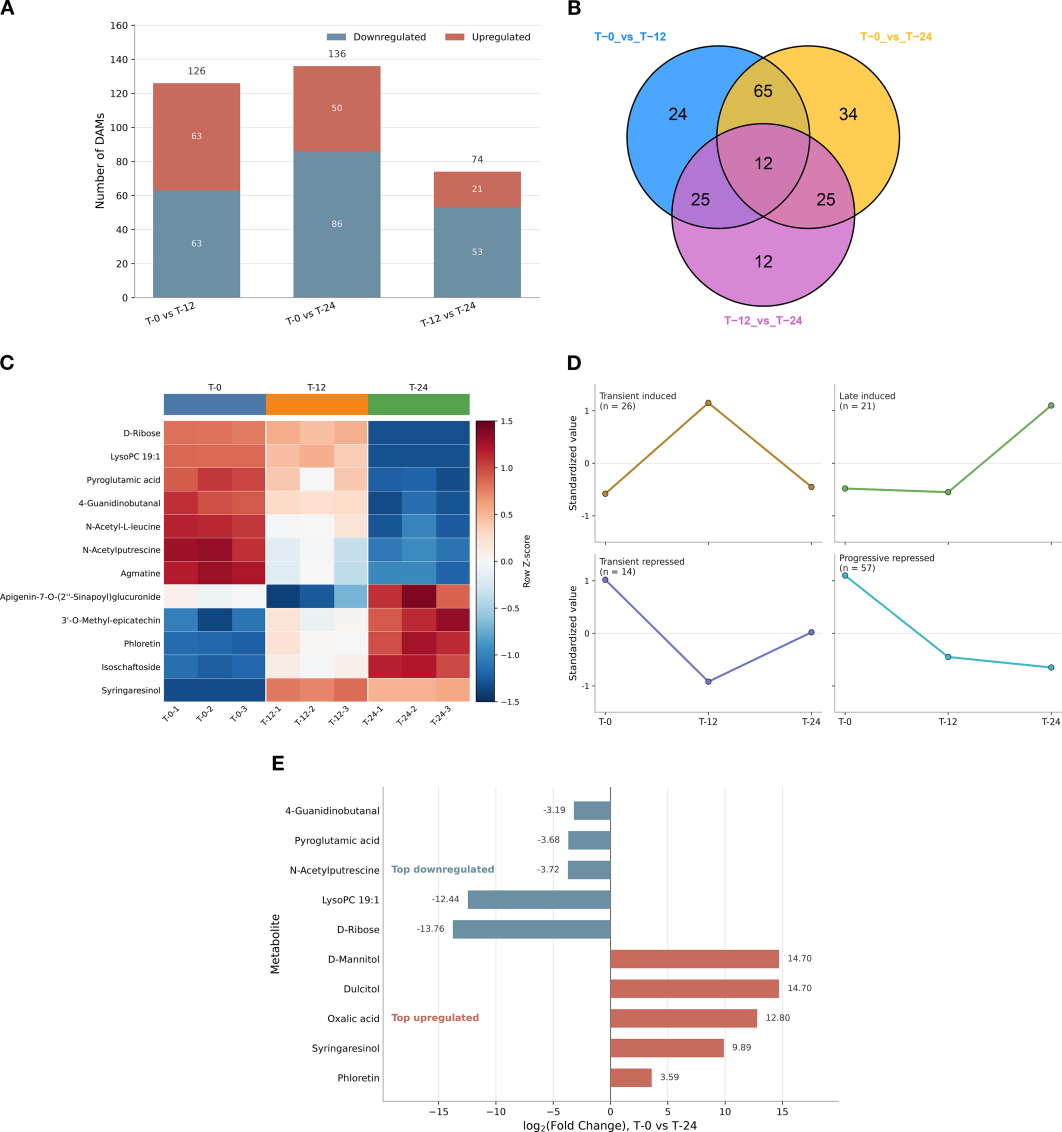
Differentially accumulated metabolites (DAMs), shared DAM profiles, and representative accumulation patterns during postharvest warm conditioning of *C. oleifera* seed-kernel tissues. **(A)** Numbers of significantly upregulated and downregulated DAMs identified in the three pairwise comparisons: T-0 vs T-12, T-0 vs T-24, and T-12 vs T-24. **(B)** Venn diagram showing the overlap of DAMs among the three pairwise comparisons, including 12 shared DAMs detected in all three contrasts. **(C)** Heatmap showing the relative accumulation patterns of the 12 shared DAMs across T-0, T-12, and T-24 samples. Values are displayed as row-scaled Z-scores of metabolite abundance. **(D)** Representative temporal accumulation patterns derived from clustering analysis of significant metabolites, including transient induced, late induced, transient repressed, and progressive repressed trajectories. Values are shown as standardized relative abundances. **(E)** Top regulated metabolites in the T-0 vs T-24 comparison, ranked according to the magnitude and direction of log_2_ fold change.

To further characterize the metabolomic response to postharvest warm conditioning, we next focused on significantly differentially accumulated metabolites (DAMs). Using VIP ≥ 1 together with fold change ≥ 2 or ≤ 0.5 as the screening criteria, we identified 126, 136, and 74 DAMs in the T-0 vs T-12, T-0 vs T-24, and T-12 vs T-24 comparisons, respectively (**Figure 7A**). The T-0 vs T-12 comparison showed a balanced structure, with 63 upregulated and 63 downregulated metabolites. By contrast, the T-0 vs T-24 comparison yielded the largest DAM set and a stronger bias toward decreases, comprising 50 upregulated and 86 downregulated metabolites. The T-12 vs T-24 comparison identified 21 upregulated and 53 downregulated DAMs. Together, these results indicate substantial metabolite remodeling during postharvest warm conditioning, with both the magnitude and direction of change varying across the time course.

To complement the comparison-specific DAM sets, we next examined their overlap across the three pairwise comparisons. Twelve DAMs were shared among all three contrasts, whereas the remaining DAMs were either comparison-specific or shared between only two contrasts (**Figure 7B**). We then visualized the relative accumulation patterns of these 12 shared DAMs across T-0, T-12, and T-24. The heatmap showed that the shared DAMs did not follow a single common trajectory, but instead displayed distinct treatment-dependent accumulation patterns across the conditioning series (**Figure 7C**). This shared DAM subset therefore points to a core, but heterogeneous, metabolite response associated with postharvest warm conditioning. To move beyond the shared DAM subset alone, we further summarized representative temporal accumulation patterns by clustering significant metabolites according to their trajectories (**Figure 7D**). Four recurrent response modes were highlighted: transient induced, in which metabolite abundance peaked at 12 h and declined by 24 h; late induced, in which accumulation became most pronounced at 24 h; transient repressed, characterized by marked depletion at 12 h followed by partial recovery at 24 h; and progressive repressed, in which metabolite levels decreased continuously across the conditioning series. These representative trajectories indicate that the metabolomic response was temporally structured rather than uniform.

We next highlighted the most strongly shifted metabolites in the T-0 vs T-24 comparison, which provided the clearest endpoint contrast in the present experiment (**Figure 7E**). Metabolites with the largest positive shifts included D-mannitol, dulcitol, oxalic acid, syringaresinol, and phloretin, whereas the strongest negative shifts were observed for D-ribose, LysoPC 19:1, N-acetylputrescine, pyroglutamic acid, and 4-guanidinobutanal. Although these metabolites span diverse biochemical classes, their coordinated redistribution is consistent with broad metabolite reorganization during postharvest warm conditioning. Together, these results show that conditioned seed-kernel tissues exhibited not only extensive DAM turnover but also a shared core DAM set and structured temporal accumulation trajectories, thereby providing a metabolite-level complement to the transcriptomic responses described above.

### 3.8 KEGG pathway enrichment of differentially accumulated metabolites during postharvest warm conditioning

**Figure 8:**
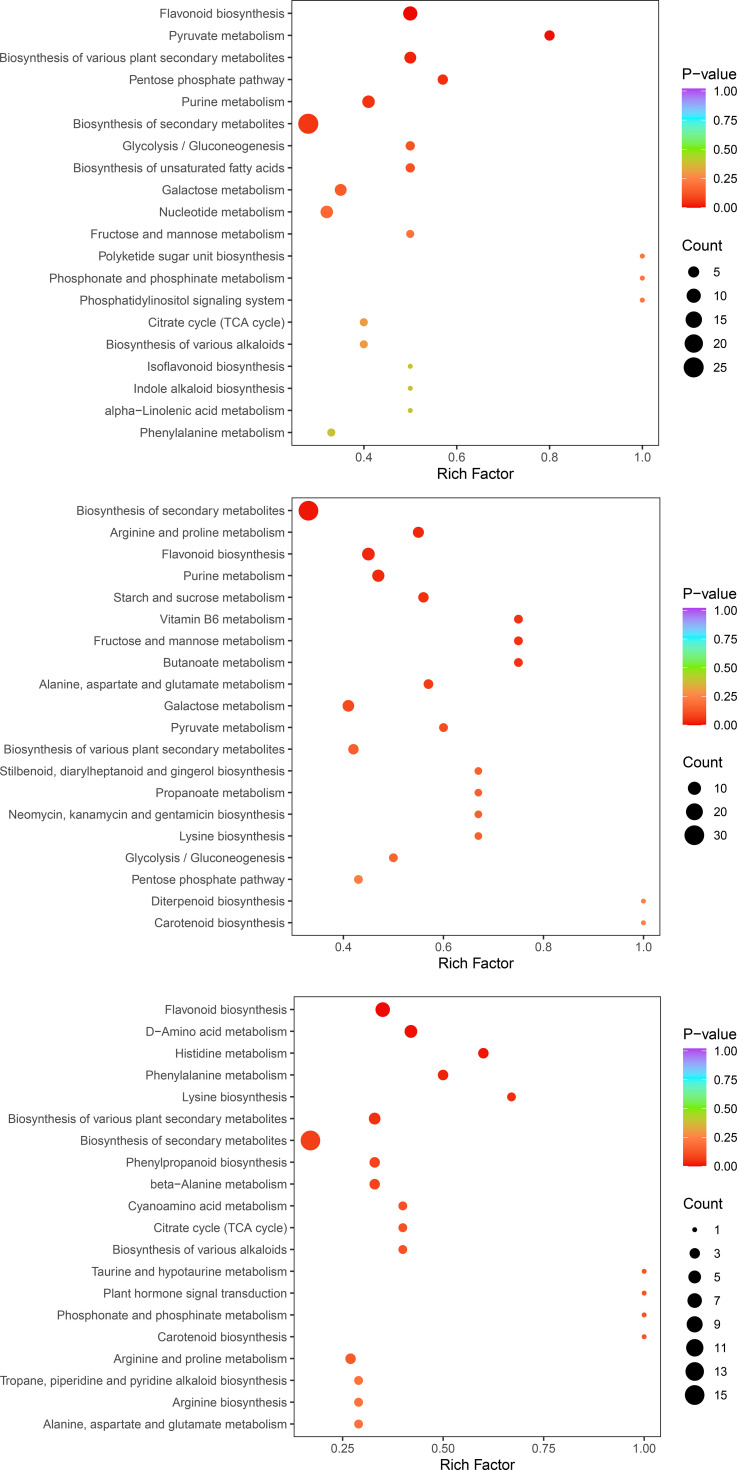
KEGG pathway enrichment analysis of differentially accumulated metabolites (DAMs) during postharvest warm conditioning of *C. oleifera* seed-kernel tissues. **(A–C)** Bubble plots showing enriched KEGG pathways for the three pairwise comparisons: T-0 vs T-12 **(A)**, T-0 vs T-24 **(B)**, and T-12 vs T-24 **(C)**. The y-axis lists enriched KEGG pathways, and the x-axis indicates the rich factor. Bubble size represents the number of DAMs mapped to each pathway (Count), and bubble color indicates enrichment significance (p-value), with smaller p-values denoting stronger enrichment.

To further interpret the biological significance of the metabolite shifts, we performed KEGG pathway enrichment analysis on the differentially accumulated metabolites (DAMs) identified in the three pairwise comparisons (**Figures 8A–C**). Across all comparisons, DAMs were repeatedly enriched in pathways related to secondary metabolism, especially biosynthesis of secondary metabolites, biosynthesis of various plant secondary metabolites, and flavonoid biosynthesis, indicating broad remodeling of specialized metabolite composition during postharvest warm conditioning (Wang et al., 2024; Ninkuu et al., 2025). Because such pathways are frequently associated with stress-responsive metabolic adjustment in plants, this enrichment pattern provides useful biochemical context for the treatment response observed here (Obata and Fernie, 2012). The early comparison (T-0 vs T-12) was additionally characterized by enrichment of pyruvate metabolism, the pentose phosphate pathway, purine metabolism, and glycolysis/gluconeogenesis, together with lipid-related pathways such as biosynthesis of unsaturated fatty acids and α-linolenic acid metabolism (**Figure 8A**) (Chen et al., 2024). This pattern suggests rapid adjustment of central carbon flow and redox-associated metabolism during the initial phase of warm conditioning. In the more prolonged comparison (T-0 vs T-24), enriched pathways extended to starch and sucrose metabolism, vitamin B6 metabolism, arginine and proline metabolism, and other amino-acid-associated pathways (**Figure 8B**), consistent with broader metabolic reconfiguration during extended treatment. Because vitamin B6 vitamers have been linked to stress-associated redox processes in plants, we interpret this enrichment as a pathway-level association rather than direct evidence of increased antioxidant capacity (Dell’Aglio et al., 2017). By contrast, the transition from 12 to 24 h (T-12 vs T-24) preferentially enriched pathways related to phenylalanine metabolism, phenylpropanoid biosynthesis, histidine metabolism, and D-amino acid metabolism, while flavonoid biosynthesis and secondary-metabolite biosynthesis remained represented (**Figure 8C**) (Ninkuu et al., 2025). Together, these results indicate that postharvest warm conditioning induced a temporally structured metabolic remodeling in *C. oleifera* seed-kernel tissues, progressing from early central metabolic adjustment to broader secondary-metabolite and amino-acid-associated remodeling. These pathway-level signatures provide an important biochemical context for the transcriptomic patterns described above.

### 3.9 Validation of squalene accumulation and HMGR abundance during postharvest warm conditioning

To biochemically support the transcriptomic and metabolomic results, we quantified squalene content and measured HMGR abundance in *C. oleifera* seed-kernel tissues after postharvest warm conditioning (**Table 1**). Relative to the control (T-0), squalene content increased to 314.3 μg/g at 12 h (T-12) and remained elevated at 309.1 μg/g at 24 h (T-24) (P < 0.05, n = 3). Given this HMGR-associated change in squalene output, we focused subsequent analyses on HMGR isoforms as the rate-limiting node together with phosphorylation-associated components; transcript-level responses of additional downstream enzymes in the squalene/sterol branch were not systematically profiled in this study. Consistently, ELISA-measured HMGR abundance increased from 10.70 U/g FW in T-0 to 13.74–14.03 U/g FW in postharvest warm conditioning samples (P < 0.05, n = 3). Relative to T-0, both comparisons showed large treatment effects for squalene accumulation and HMGR abundance, as reflected by the corresponding *Hedges’ g* values in **Table 1**. Collectively, these biochemical measurements corroborated a warm conditioning-associated increase in squalene levels accompanied by elevated HMGR abundance in seed-kernel tissues.

### 3.10 Analysis of the correlation between the transcriptome and metabolome

To examine the transcriptional behavior of the HMGR family during postharvest warm conditioning, we analyzed HMGR-related transcripts in the assembled *C. oleifera* transcriptome (**Table 2**). Four HMGR transcripts were detected, consistent with a recent report describing four *CoHMGR* genes in this species (Gu et al., 2025). Among them, *HMGR-2* (TRINITY_DN57861_c3_g3) showed the highest absolute TPM values across the series, but its abundance declined progressively from 2.92 ± 2.16 at T-0 to 1.12 ± 0.37 at T-12 and 0.34 ± 0.28 at T-24. By contrast, *HMGR-1* (TRINITY_DN57861_c3_g1) remained a low-abundance transcript overall, although it showed a transient relative increase at 12 h (0.15 ± 0.09) compared with T-0 (0.02 ± 0.03) and T-24 (0.05 ± 0.04). *HMGR-3* and *HMGR-4* were also detected at low levels and did not show a robust induction pattern.

**Table 2:** Expression levels (TPM ± SD) of *HMGR* isoforms in *C. oleifera* seed-kernel tissues during postharvest warm conditioning.

Isoform ID	Transcript ID	T-0 (mean ± SD)	T-12 (mean ± SD)	T-24 (mean ± SD)
*HMGR-1*	TRINITY_DN57861_c3_g1	0.02 ± 0.03	0.15 ± 0.09^a^	0.05 ± 0.04
*HMGR-2*	TRINITY_DN57861_c3_g3	2.92 ± 2.16	1.12 ± 0.37	0.34 ± 0.28
*HMGR-3*	TRINITY_DN67520_c1_g4	0.91 ± 0.63	0.67 ± 0.43	0.42 ± 0.24
*HMGR-4*	TRINITY_DN76432_c0_g1	0.14 ± 0.24	0.04 ± 0.07	0.18 ± 0.31

TPM values are expressed as mean ± SD (n = 3 biological replicates). Superscript letters (^a^) indicate statistically significant induction compared with T-0 (P < 0.05, one-way ANOVA + Tukey’s HSD).

Because HMGR encodes the rate-limiting enzyme of the mevalonate pathway (Yang et al., 2022), these data are relevant for defining the detectable HMGR family landscape under postharvest warm conditioning. However, all four isoforms were present at low absolute TPM levels, and several showed substantial replicate variability. Accordingly, the observed transcript patterns are more appropriately interpreted as low-abundance isoform profiles than as definitive evidence of isoform-specific regulation. In this context, the present transcriptome dataset is informative for describing relative HMGR-family behavior, but it does not resolve the contribution of individual isoforms to total HMGR abundance or squalene accumulation. Clarifying these isoform-specific roles will require targeted validation at both the transcript and protein levels.

**Figure 9:**
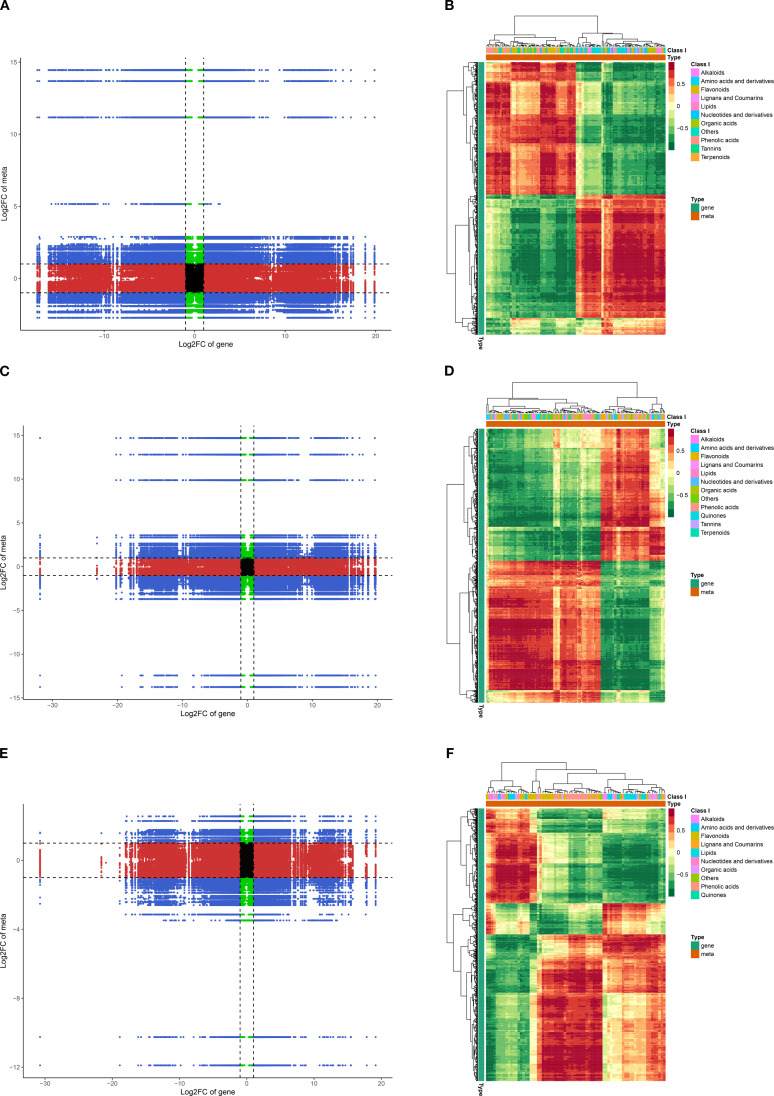
Correlation analysis between transcriptome and metabolome during postharvest warm conditioning. **(A, C, E)** Nine-quadrant plots summarizing concordance between DEG and DAM changes for T-0 *vs* T-12 **(A)**, T-0 *vs* T-24 **(C)**, and T-12 *vs* T-24 **(E)**. The x-axis shows log_2_ fold change of genes and the y-axis shows log_2_ fold change of metabolites. Dashed lines indicate the differential-change cutoffs used to assign quadrants. Quadrants 3 and 7 denote concordant changes (both gene expression and metabolite abundance increase or decrease together), quadrants 1 and 9 denote discordant changes (opposite directions), quadrants 2/4/6/8 indicate that only one layer passes the cutoff (gene-only or metabolite-only), and quadrant 5 indicates no differential change in either layer. **(B, D, F)** Hierarchical clustering heatmaps of pairwise correlation coefficients between DEGs (rows) and DAMs (columns) for T-0 *vs* T-12 **(B)**, T-0 *vs* T-24 **(D)**, and T-12 *vs* T-24 **(F)**. Color scale indicates correlation strength and direction (red, positive; green, negative). Metabolites are annotated by chemical class (top bar), providing an overview of coordinated transcript–metabolite associations during postharvest warm conditioning at 35 °C.

We next integrated transcriptome and metabolome datasets using nine-quadrant analysis (**Figures 9A, C, E**). Across all three pairwise comparisons, the distributions of points across concordant and discordant quadrants indicated that postharvest warm conditioning triggered both coordinated and decoupled responses between gene expression and metabolite accumulation. In other words, some transcript–metabolite pairs changed in the same direction, whereas others changed in opposite directions or at only one molecular layer. This overall pattern suggests that the warm conditioning response involved structured but nonuniform reorganization across the two datasets, rather than a simple one-to-one coupling between transcripts and metabolites. The corresponding correlation heatmaps (**Figures 9B, D, F**) further revealed widespread positive and negative transcript–metabolite associations across the three comparisons. These matrices did not reveal a single dominant relationship pattern; instead, they showed that HMGR-associated metabolic responses were embedded within a broader network involving multiple metabolite classes, including amino acids and derivatives, flavonoids, lipids, nucleotides and derivatives, organic acids, phenolic acids, tannins, and terpenoids. Thus, the integrated analysis supports the view that postharvest warm conditioning reshaped transcript–metabolite relationships at the network level. Notably, key upstream MVA-pathway precursor intermediates, including acetyl-CoA, mevalonate, and IPP/DMAPP, were not detected in the present broadly targeted metabolomic dataset. Accordingly, the current results do not permit direct assessment of precursor-pool dynamics, and the observed HMGR-associated transcriptional and metabolic changes are more appropriately interpreted as evidence of pathway engagement and downstream metabolic reorganization rather than direct shifts in precursor abundance. Targeted quantification of these intermediates will be important in future work to define precursor-pool dynamics more explicitly.

At the candidate-metabolite level, HMGR showed notable associations with several representative metabolites. In quadrant 3, where both transcript abundance and metabolite accumulation increased, HMGR was positively associated with 4-pyridine-O-glucoside, a vitamin B6-related metabolite previously linked to stress-associated redox processes (Havaux et al., 2009). By contrast, in quadrant 9, where HMGR increased while metabolite abundance decreased, HMGR showed negative associations with metabolites such as D-fructose and vitexin glucoside. These opposite-direction relationships are more consistent with redistribution of carbohydrate- and secondary-metabolite pools during warm conditioning than with direct positive coupling (Obata and Fernie, 2012). Accordingly, the integrated analysis suggests that HMGR operates within a broader stress-responsive metabolic context rather than acting in isolation. Overall, the transcriptome–metabolome association analysis indicates that HMGR-centered responses during postharvest warm conditioning were accompanied by extensive metabolic-network reorganization. When considered together with the biochemical validation presented above, these data support a consistent interpretation in which *HMGR-1* was the most strongly inducible transcript among the HMGR family members under postharvest warm conditioning. Nevertheless, these results establish correlation rather than direct causality, and the mechanistic role of HMGR in the postharvest response network will require further validation at the protein and post-translational levels, including interaction- and phosphorylation-oriented analyses (Falcone Ferreyra et al., 2012; Tholl, 2015; Shen et al., 2016).

### 3.11 Full-length *CoHMGR* cloning, candidate phosphosite prediction, and coordinated transcriptional responses during postharvest warm conditioning

**Figure 10:**
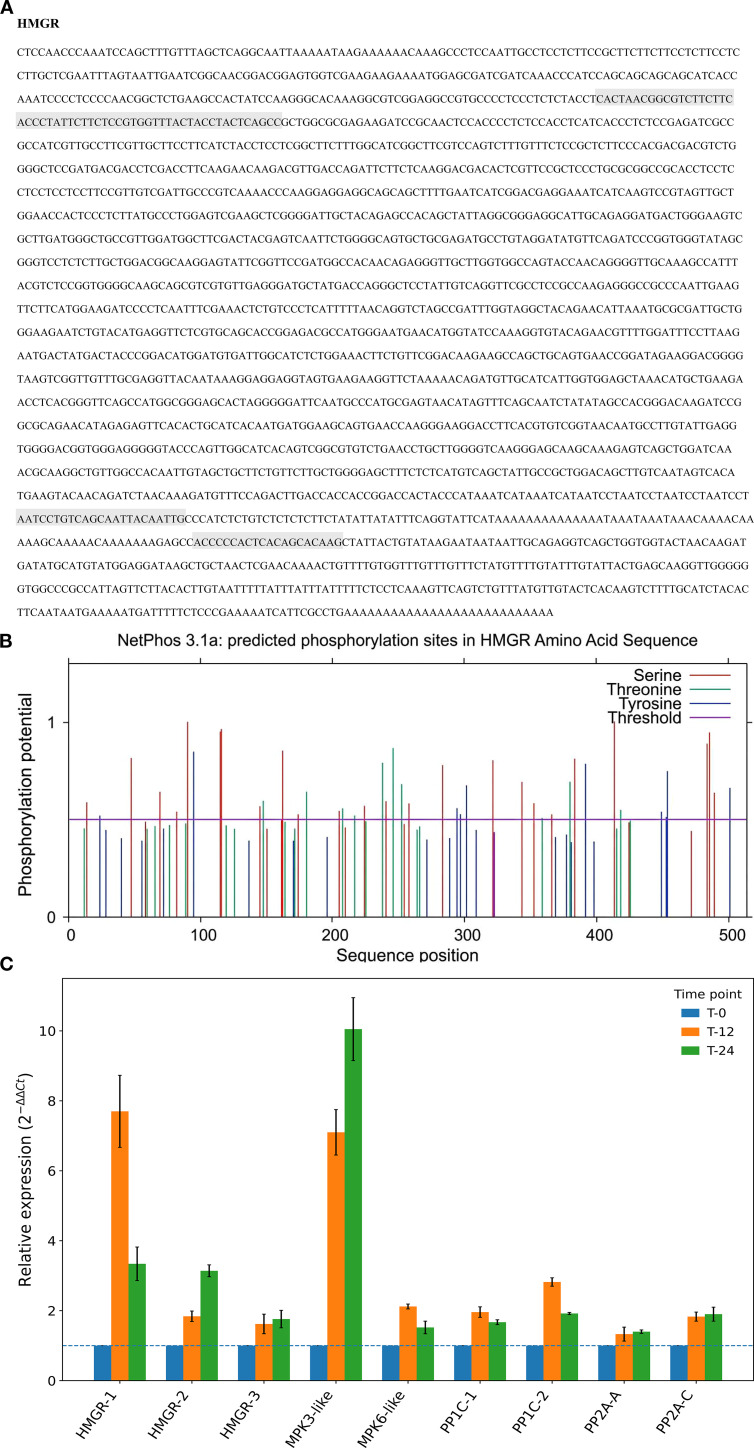
Full-length cloning of *CoHMGR*, in silico prediction of candidate phosphorylation sites, and RT–qPCR validation of warm conditioning-responsive transcripts in *C. oleifera* seed-kernel tissues. **(A)** Full-length *CoHMGR* cDNA obtained by rapid amplification of cDNA ends (RACE). **(B)** NetPhos 3.1 prediction of candidate phosphorylation sites along the *CoHMGR* amino-acid sequence. Predicted Ser, Thr, and Tyr residues are shown by sequence position with their corresponding scores, and the horizontal line indicates the default prediction threshold. Predicted sites were concentrated primarily on Ser and Thr residues. Notable candidate sites included Ser47, Thr208, and Ser217, whereas Ser116 (0.960), Thr246 (0.861), and Thr238 (0.787) showed the highest prediction scores. **(C)** RT–qPCR validation of RNA-seq–selected genes in seed-kernel tissues from T-0 and warm-conditioned samples (35 °C; T-12 and T-24). Relative transcript levels of *HMGR-1*, *HMGR-2*, *HMGR-3*, *MPK3-like*, *MPK6-like*, *PP1c-1*, *PP1c-2*, *PP2A-A*, and *PP2A-C* were normalized to SAND and expressed as fold change relative to T-0 (2^−ΔΔCt; T-0 = 1). Bars represent mean ± SD (n = 3 biological replicates). The dashed line marks the baseline. *HMGR-4* was not detected.

In this study, full-length *CoHMGR* cDNA was obtained by rapid amplification of cDNA ends (RACE) (**Figure 10A**). NetPhos 3.1 predicted multiple putative phosphorylation sites along the *CoHMGR* sequence, with signals concentrated mainly on serine (Ser) and threonine (Thr) residues (**Figure 10B**). Notable candidate sites included Ser47, Thr208, and Ser217, whereas Ser116, Thr246, and Thr238 showed the highest prediction scores (0.960, 0.861, and 0.787, respectively). Several sites were further assigned as potential targets of kinases such as PKC, GSK3, p38 MAPK, and CDK5, suggesting that *CoHMGR* may respond to phosphorylation-linked signaling inputs (Zhu et al., 2024).

HMGR is widely recognized as a phosphoregulated enzyme, with phosphorylation and dephosphorylation influencing catalytic status and downstream isoprenoid metabolism (Antolín-Llovera et al., 2011; Leivar et al., 2011). In *Arabidopsis thaliana*, PP2A interacts with the conserved N-terminal region of HMGR through its B″ regulatory subunit and contributes to HMGR phosphoregulation and turnover (Leivar et al., 2011). Additional studies indicate that HMGR regulation may involve multiple phosphorylation sites beyond the conserved catalytic-domain residue Ser577, pointing to context-dependent control under changing environmental conditions (Antolín-Llovera et al., 2011; Robertlee et al., 2017). By comparison, evidence for PP1 involvement in plant HMGR regulation remains limited, although PP1 is a major Ser/Thr phosphatase with established roles in ABA signaling, stomatal movement, and kinase-associated processes (Takemiya and Shimazaki, 2010; Jo et al., 2025).

Consistent with this broader framework, our transcriptome dataset identified several phosphatase-related candidates, including *PP2A-C* (TRINITYDN60447_c1_g1), *PP2A-A* (TRINITYDN65830_c0_g1), and a PP1 catalytic subunit (TRINITYDN67318_c1_g3), all of which showed increased transcript abundance under postharvest warm conditioning. In parallel, RT–qPCR analysis of nine selected warm-conditioning-responsive targets confirmed trends consistent with the RNA-seq data (**Figure 10C**). Among the HMGR isoforms, *HMGR-1* showed the strongest induction, peaking at 7.7-fold at 12 h and declining to 3.4-fold at 24 h, whereas *HMGR-2* and *HMGR-3* displayed more moderate increases (~1.6–3.2-fold). *MPK3-like* and *MPK6-like* transcripts were also induced, with *MPK3-like* showing the larger dynamic range (~7.1–10.1-fold versus ~1.5–2.1-fold). Phosphatase-related genes increased more modestly, with *PP1c-1/PP1c-2* rising by ~1.7–2.8-fold and *PP2A-A/PP2A-C* by ~1.3–1.9-fold. The corresponding pairwise comparisons against T-0 also yielded predominantly moderate-to-large *Hedges’ g* values for the major induced transcripts, consistent with the magnitude of the RT–qPCR response (**Table 3**).

**Table 3:** Fold changes and effect sizes of RT–qPCR-validated transcripts during postharvest warm conditioning in *C. oleifera* seed-kernel tissues.

Gene	Fold change (T-12 vs T-0)	*Hedges’ g* (T-12 vs T-0)	Fold change (T-24 vs T-0)	*Hedges’ g* (T-24 vs T-0)
*HMGR-1*	7.71	17.59	3.36	9.62
*HMGR-2*	1.84	7.87	3.15	23.29
*HMGR-3*	1.62	3.13	1.75	4.29
*MPK3-like*	7.12	25.31	10.15	28.17
*MPK6-like*	2.15	29.33	1.52	3.93
*PP1c-1*	1.95	9.11	1.66	13.76
*PP1c-2*	2.83	26.83	1.90	47.05
*PP2A-A*	1.35	2.42	1.41	8.59
*PP2A-C*	1.85	8.44	1.91	6.05

Values are based on RT–qPCR fold changes relative to T-0 (2^−ΔΔCt; T-0 = 1). Effect sizes for pairwise comparisons against T-0 were calculated as *Hedges’ g* using dCt values, because the normalized fold-change output fixes T-0 at 1 and is therefore unsuitable for variance-based effect-size estimation. *HMGR-4* was not detected in this assay.

Together, the predicted *CoHMGR* phosphosites, the induction of MAPK- and phosphatase-related transcripts, and the concordant RT–qPCR validation support a phosphorylation-associated interpretive framework for the warm conditioning response in *C. oleifera* seed-kernel tissues. In particular, Ser47 and Thr208 emerged as candidate residues of interest because they are positioned near the N-terminal regulatory region and catalytic domain, respectively, and coincide with the induction of MPK3/*MPK6-like* components during treatment, consistent with the established role of MAPK cascades in plant heat- and stress-responsive signaling (Asai et al., 2002). Further protein-level and phospho-state analyses, together with finer early time-course sampling beyond the present 0-, 12-, and 24-h design, will be required to resolve the earliest signaling and transcriptional transitions underlying these coordinated responses.

## 4 Conclusions

This study integrated transcriptomics, metabolomics, and targeted biochemical measurements to characterize the early postharvest warm conditioning response in *C. oleifera* seed-kernel tissues. Incubation at 35 °C and 95% RH was accompanied by increased squalene content and elevated HMGR abundance, together with broad transcriptional reprogramming involving MVA-associated genes and multiple phosphorylation-related components, including *MPK3/MPK6*-like kinases and PP1/PP2A subunits. Isoform-resolved analysis indicated that *HMGR-2* predominated at baseline, whereas *HMGR-1* was the most strongly inducible HMGR transcript under treatment. Together, these datasets provide an interpretive framework linking postharvest warm conditioning to HMGR/MVA-associated outputs and identify phosphorylation-related candidates for future protein-level and phospho-state validation.

## Data Availability

The RNA-seq datasets generated and analyzed in this study have been deposited in the NCBI Sequence Read Archive (SRA) under BioProject accession number PRJNA1355314. Additional data supporting the findings of this study are included in the article and its Supplementary Material. Further information is available from the corresponding author upon reasonable request.
