# Correction: Postharvest warm conditioning associates with HMGR abundance, squalene accumulation, and MAPK/phosphatase transcriptional responses in *Camellia oleifera*

**DOI:** 10.3389/fpls.2026.1867499

**Published:** 2026-05-11

**Authors:** Frontiers Production Office

**Affiliations:** Frontiers Media SA, Lausanne, Switzerland

**Keywords:** *Camellia oleifera*, HMGR, MAPK signaling, postharvest warm conditioning, protein phosphatase, squalene accumulation

The affiliation for reviewer “Habtamu Kefale” was incorrectly stated as “Hainan Academy of Agricultural Sciences, China”. The correct affiliation is “Chinese Academy of Tropical Agricultural Sciences, China”.

The original version of this article has been updated.

