# Supplementary material for: Postharvest warm conditioning associates with HMGR abundance, squalene accumulation, and MAPK/phosphatase transcriptional responses in Camellia oleifera
Source: Front Plant Sci. 2026 Apr 23;17:1804924. doi: 10.3389/fpls.2026.1804924 (PMC13149285; doi:10.3389/fpls.2026.1804924)
Supplement: Supplementary file 1 [file DataSheet1.pdf]

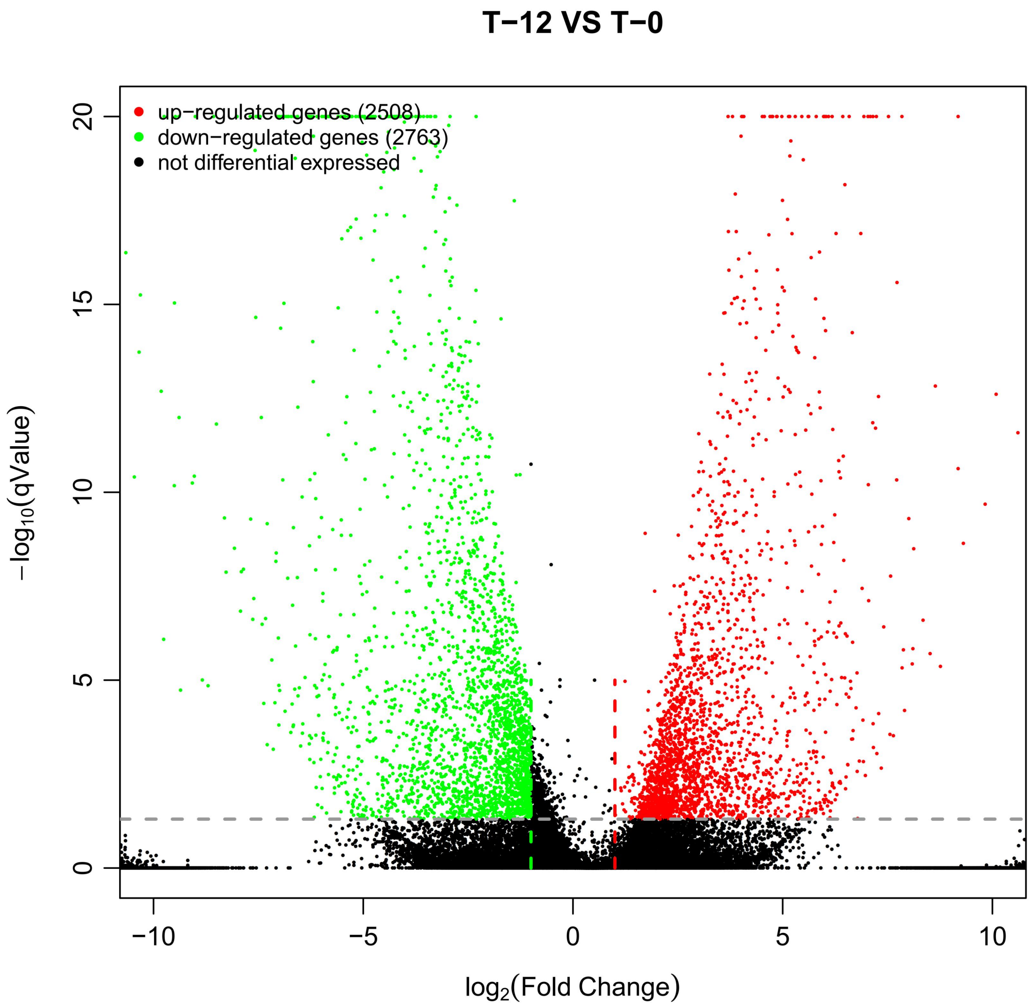
T−12 VS T−0
up−regulated genes (2508)
down−regulated genes (2763)
not differential expressed
−log10(qValue)
log2(Fold Change)
0
5
10
15
20
−10
−5
0
5
10

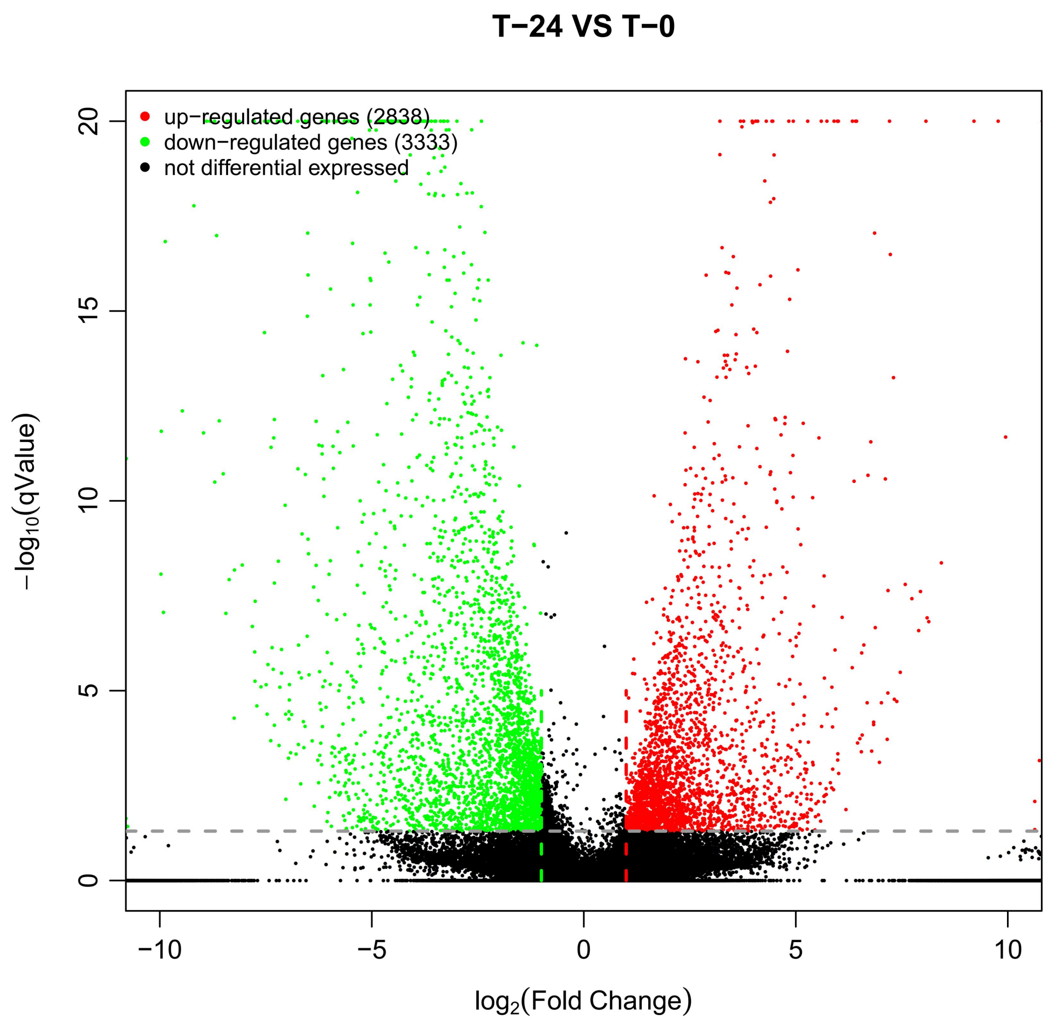
T−24 VS T−0
up−regulated genes (2838)
down−regulated genes (3333)
not differential expressed
−log10(qValue)
log2(Fold Change)
0
5
10
15
20
−10
−5
0
5
10

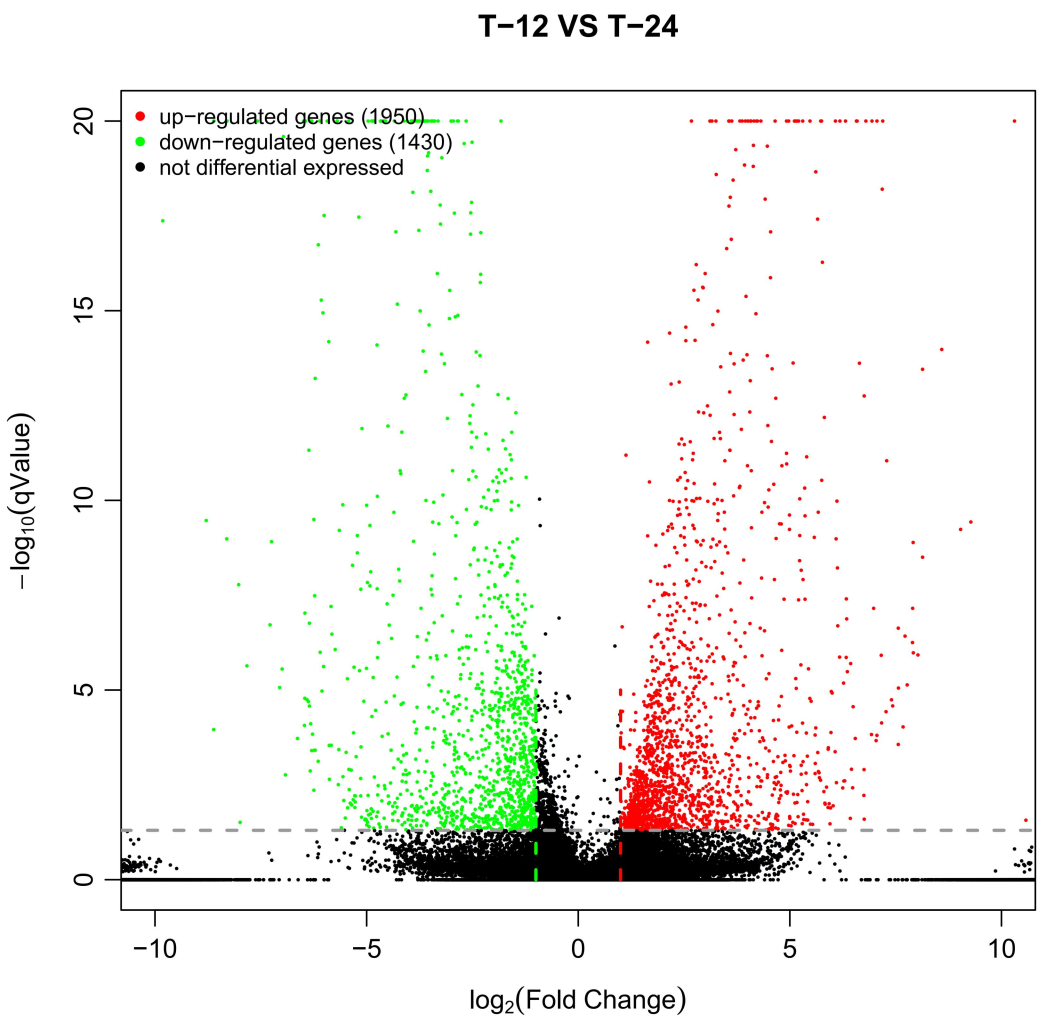

Fig. S1. Volcano plots of differentially expressed genes (DEGs) in pairwise transcriptomic comparisons during postharvest warm conditioning of Camellia oleifera seed-kernel tissues.

(A) T-12 vs T-0.

(B) T-24 vs T-0.

(C) T-12 vs T-24.

Red dots indicate upregulated genes, green dots indicate downregulated genes, and black dots indicate genes without significant differential expression. DEGs were defined using a q-value < 0.05 and an absolute fold change > 2.
